# Discovery and Preclinical Development of Orally Active Small Molecules that Exhibit Highly Selective Follicle Stimulating Hormone Receptor Agonism

**DOI:** 10.3389/fphar.2020.602593

**Published:** 2021-01-14

**Authors:** Selva Nataraja, Henry Yu, Joie Guner, Stephen Palmer

**Affiliations:** ^1^ TocopheRx, Inc., Groton, MA, United States; ^2^ Center for Drug Discovery, Baylor College of Medicine, Houston, TX, United States

**Keywords:** follicle stimulating hormone, oral FSHR allosteric agonist, G-protein coupled receptor, infertility treatment, follicular maturation, oocyte viability

## Abstract

An orally active follicle stimulating hormone receptor allosteric agonist would provide a preferred treatment for over 16 million infertile women of reproductive age in low complexity methods (ovulation induction-intrauterine insemination) or in high complexity methods (controlled ovarian stimulation-*in vitro* fertilization). We present two oral follicle stimulating hormone receptor allosteric agonist compounds that have the desired pharmacology, drug metabolism, pharmacokinetics, and safety profile for clinical use. These molecules provide a single agent suitable for ovulation induction-intrauterine insemination or controlled ovarian stimulation-*in vitro* fertilization that is more convenient for patients and achieves similar preclinical efficacy as rec-hFSH. TOP5668, TOP5300 were evaluated *in vitro* in Chinese hamster ovary cells transfected with individual glycoprotein receptors measuring cAMP (FSHR, LH/CGR, thyroid stimulating hormone receptor). TOP5668 was found to have solely follicle stimulating hormone receptor allosteric agonist activity while TOP5300 was found to have mixed follicle stimulating hormone receptor allosteric agonist and LHR-AA activity. Both compounds stimulated concentration-dependent increases in estradiol production from cultured rat granulosa cells in the presence or absence of low dose rec-hFSH, while only TOP5300 stimulated testosterone production from rat primary Leydig cells. In pooled human granulosa cells obtained from patients undergoing controlled ovarian stimulation-*in vitro* fertilization, TOP5300 stimulated 7-fold greater maximal estradiol response than rec-hFSH and TOP5668 was 10-fold more potent than TOP5300. Both TOP5300 and TOP5668 stimulated follicular development in immature rat to the same efficacy as recombinant follicle stimulating hormone. In mice treated with TOP5300, in the presence of low dose of follicle stimulating hormone, there were no differences in oocyte number, fertilization rate, and hatched blastocyst rate in mice with TOP5300 and low dose follicle stimulating hormone vs. reference proteins pregnant mare serum gonadotropin or high dose rec-hFSH. ADME/PK and safety profiles were favorable. In addition, there was no appreciable activity on thyroid hormones by TOP5300 in 14-days toxicological study in rat or dog. The selected lead compound, TOP5300 stimulated a more robust increase in estradiol production from granulosa-lutein cells from women with polycystic ovarian syndrome patient compared to rec-hFSH. Conclusions: Two novel oral FSHR allosteric agonist, TOP5668 and TOP5300, were found to mimic the biological activity of rec hFSH in preclinical studies. Both compounds led to folliculogenesis and superovulation in rat and mice. Specifically, TOP5300 led to a similar number of ovulated oocytes that fertilized and developed into hatched blastocysts in mice when compared to rec-hFSH. The safety profile demonstrated lack of toxicity.

## Introduction

Meaningful changes in therapeutic regimens for low complexity (ovulation induction-intrauterine insemination; OI-IUI) and higher complexity controlled ovarian stimulation-*in vitro* fertilization [COS-IVF]) cycles have followed innovations of follicle stimulating hormone (FSH) (Grainger et al., 2013). In the normal ovarian cycle, FSH is secreted by the pituitary gland and stimulates ovarian follicular maturation through binding to its Gs-protein coupled receptor expressed on granulosa cells. Receptor binding triggers a classic pathway response of cAMP dependent protein-kinase A and CREB (cyclic AMP Response Element Binding Protein) phosphorylation that stimulates aromatization of thecal cell-derived androgen and provides a positive feedback signal to the female hypothalamus and pituitary of readiness for a follicle to ovulate (Jiang et al., 2015). In the past, glycosylation sites have been added to recombinant FSH or inserted into FSH to generate a longer half-life agonist (Fares et al., 1992). However, the resulting corifollitropin has not consistently yielded measurable improvements in clinical pregnancy rates (Cozzolino et al., 2019). The patient experience with recombinant corifollitropin offers the first opportunity to gauge treatment preference of patients for fewer injections and less stress associated with controlled ovarian stimulation protocols utilizing FSH (Requena et al., 2013). Administration of only corifollitropin, without supplementary daily injections of FSH, produced less discomfort than daily injections. Among women that had a previous COS-IVF cycle with daily injections of FSH, there was a 75% preference for corifollitropin if no additional daily FSH was required to supplement corifollitropin. It is reasonable to expect that oral administration of allosteric agonists of the FSH receptor (FSHR-AA), without supplemental injectable products, will exceed the convenience measured for corifollitropin, and have less of a negative impact on the endometrium than current orally available clomiphene (Chen et al., 2016; Mehdinejadiani et al., 2019).

### Anticipated Patient Benefit of FSHR-AA

Orally active gonadotropin agonists are anticipated to provide measurable benefits for patients that include: a) faster elimination of small molecules from blood (predicted 5–9 h) than injectable high sialic acid-glycosylated gonadotropins (9–24 h) (le Contonnec et al., 1994; Wide and Eriksson, 2013; Bousfield et al., 2018; Wide and Eriksson, 2018; Riccetti et al., 2019) or clomiphene (72 + *h*) (Ghobadi et al., 2009); b) greater predictability of individual patient responses to the same molecule as patients transition from low dose, low complexity OI-IUI cycles into higher dose, COS-IVF cycles; c) direct action of FSHR-AA on FSHR, compared to CC and aromatase inhibitors which depend on the integrity of the hypothalamic-pituitary-ovarian axis; d) absence of compromise to endometrial implantation when compared to CC (Chen et al., 2016; Mehdinejadiani et al., 2019) and e) small molecule gonadotropin agonists work at the FSH receptor independent of the glycosylation of endogenous FSH that varies over the menstrual cycle (Bousfield et al., 2018; Wide and Eriksson, 2018) and among glycoprotein hormone preparations of FSH (Wide and Eriksson, 2013; Riccetti et al., 2019). It is anticipated that these benefits can deliver improvements in stimulation outcomes and as a result, reduced time for patients to achieve a live birth.

### Innovations of FSH Receptor Structure

Resolving the crystal structure of FSH bound to the orthosteric leucine-rich-repeats in the extracellular domain of the 7-transmembrane, G-protein coupled FSH receptor revealed that activation of the receptor occurred through release of an inverse agonist domain in the ectodomain, and subsequent release of a monomer of receptor from the basal state trimer (Jiang et al., 2012; Jiang et al., 2014a). These structures provided a logical mechanism for small molecules to behave as allosteric agonists that could not have been possible in the previously proposed models wherein the active signaling complex is created by a dimer formed from two monomers (Fan and Hendrickson, 2007). In this updated mechanism, binding of FSH to its receptor induces an intramolecular “shift” of the inverse agonist tethered domain (residues 296-331), that positions FSH β-subunit loop-2 for a “grip” by sulfotyrosine (FSHR-sTyr335). The “shift and grip” increases accessibility of a pocket suitable for entry of allosteric modulators near the extracellular surface of transmembrane (TM) helices (Ulloa-Aguirre and Zariñán, 2015). The proposed allosteric binding pocket of FSHR is surrounded by the seven transmembrane helices, capped by the extracellular loops, resembles the position of the allosteric pocket for β-adrenergic receptor (Liu et al., 2019), and offers an alternative mechanism for small molecules to displace the FSHR inverse agonist domain (Jiang et al., 2014b). According to this updated model, an allosteric agonist can independently bind to the allosteric binding pocket in the transmembrane domains and “shift” the inverse agonist away from the transmembrane domain without requirement for a subsequent “grip” of ligand by the sulfotyrosine hinge domain.

Among the FSHR allosteric modulators evaluated to date (Palmer et al., 2005; Yanofsky et al., 2006; Arey et al., 2008; Van Stratten and Timmers, 2009; van Koppen et al., 2013), none had combined sufficient potency as FSHR agonists, oral bioavailability or adequate safety profiles (Gerrits et al., 2016) suitable for human infertility treatments (reviewed) (Nataraja et al., 2021). Although convenience is a tangible benefit for patients, small molecule agonists may also generate unique mechanistic cellular responses that cannot be achieved with protein ligands. Recently, one chemical series of FSHR allosteric modulators demonstrated a three-fold increase in FSH receptor recycling compared to rec-hFSH (Sposini et al., 2020). In this report, we have described a pair of dihydropyrazoles that have been evaluated for relevant preclinical pharmacologic profiling to characterize them as FSHR allosteric agonists. They are potent, selective stimulators of ovarian follicular growth through the FSHR with an exceptional safety profile that offers an alternative to ovarian stimulation with recombinant or urinary FSH biotherapeutics. Our structure-activity guided drug discovery, combined with preclinical monitoring of thyroid stimulating hormone receptor (TSHR) activity, allowed us to develop novel FSHR-AA suitable for use in future human infertility treatments.

## Materials and Methods

### Reagents

DMEM: F-12, fetal bovine serum (FBS) and all other cell culture solutions were procured from Invitrogen (Carlsbad, CA, United States). TOP5300 and TOP5668 were synthesized at Chempartner (Shanghai, China) as part of the hit-to-lead program managed by TocopheRx. Structure and purity of the compounds were confirmed by NMR, LC/MS and HPLC. The purity of the compound employed for the study ranged from 95–97%. Recombinant human FSH (rec-hFSH) and human chorionic gonadotropin (hCG) were bulk preparations of Merck KGaA (follitropin-α, choriogonadotropin-α, Darmstadt, Germany). Pregnant mare serum gonadotropin (PMSG) and other fine chemicals were obtained from Sigma-Aldrich (St; Louis, MO, United States). Cell culture plates and dishes were purchased from Corning Inc (Corning, NY, United States). Diethylstilbesterol (DES) time release pellets and trochar were obtained from Innovative Research of America (Sarasota, FL, United States).

### Stable CHO Cell Lines Used for the Primary Evaluation of the Activity of FSHR-AA

Chinese hamster ovary (CHO) cells transfected with hFSHR, hLHR or hTSHR were grown as described previously (Kelton et al., 1992; Persani et al., 1993; Yu et al., 2014). In brief, CHO-hFSHR and CHO-hTSHR were maintained in DMEM/F12 + 10% FBS in T150 culture flasks. The cells were cultured until they reached 70–80% confluence (three days) and were then used for assays or passaged. CHO-hLHR (van de Lagemaat et al., 2009; Sriraman et al., 2014) was cultured in MEM alpha (++) +10% FBS and G418, and passaged every three days. CHO-hFSHR or −hLHR or −hTSR cells (2,500/well) were plated in 5 µL of phenol red free DMEM/F12 + 1% FBS in 384 well solid white low volume plates (Greiner 784075) by Multidrop. Compounds were tested in the presence or absence of an EC20 concentration of hFSH (0.16 pM), hCG (0.2 pM) or hTSH (13 pM), in their corresponding assays. Varying concentrations of protein ligand, or TOP5300 or TOP5668 were added to the cells in 5 μL media with 400 mM IBMX. The plates were incubated at 37°C for 1 h. After the incubation period, cAMP was measured by HTRF^®^ (homogeneous time-resolved fluorescence) assay (CisBio, Bedford, MA). In brief, conditioned media were incubated with cryptate-labeled cAMP antibody and d2-labeled cAMP in lysis buffer for 1 h. Free cAMP produced by cells competes with cAMP-d2 for binding to the anti-cAMP cryptate. An increase in cellular cAMP leads to a decrease in fluorescence resonance energy transfer, which is detectable as a decrease in the emission at 665 nm, measured by Envision fluorescence reader (PerkinElmer, Waltham, MA). The readout was calculated fluorescence ratio (665/620 nm). Values given in percent (%) indicate the percent response at a certain concentration of agonist relative to the maximum response of the protein ligand standard.

### Primary Cell Cultures of Rat Granulosa Cells or Leydig Cells

#### FSHR Activity

All animals were maintained and handled under IACUC approved procedures at the respective institutions providing contracted service for TocopheRx, following procedures previously approved by local (MA, United States) IACUC reviews (Sriraman et al., 2014; Yu et al., 2014). Ovarian follicular granulosa cells were isolated from rats implanted with DES pellets (1 mg/day; 7-days release) at ChemPartner (Shanghai, China). The pellets were implanted subcutaneously on 22 day old rats with trochar, employing aseptic techniques after isoflurane anesthesia. Animals were euthanized on post-op day 3, ovaries were excised, cleaned, and granulosa cells were isolated by repeated disruption of the follicle as described previously. Granulosa cells isolated from four to five rats were used to culture one 96-well plate and an equal number of cells was plated in each well. The cells were cultured overnight at 37°C in media containing 5% FBS.

After overnight culture, the media was replaced with serum free media containing 0.1% BSA and 100 nM androstenedione. The cells were treated with rec-hFSH or TOP5300 and TOP5668 in the presence or absence of low dose FSH (EC_20_). A dose response curve with a 4-fold dilution of rec-hFSH starting at highest concentration of 5 nM and a two-fold dilution of small molecule FSHR-AA starting at highest concentration of 10 μM at eight different concentrations was tested. After 24 h of treatment, supernatant was collected and estradiol concentration was determined by ELISA (DRG, Mountainside, NJ, United States). The optical densities of the estradiol ELISA were measured by Spectramax (Sunnyvale, CA, United States). The values were analyzed in Microsoft Excel and Graphpad Prism (San Diego, CA, United States).

#### LHR Activity

Testes were obtained from 21 day old male SD rats, decapsulated into DMEM/F12 media, placed into a spinner flask containing pre-warmed 25 ml digestion media (DMEM/F12 no phenol red + 1 mg/ml Collagenase), and stirred at 37°C in 5% CO2 incubator for 10–15 min (Hedger et al., 1990). The digestion supernatant was passed through a 70 um cell strainer into a 50 ml tube, and the digestion was stopped with 0.1% BSA + 0.1 mg/ml trypsin soybean inhibitor) in 50 ml. The digested cells were centrifuged at 800 rpm for 5 min, the supernatant discarded and the cell pellet resuspend in 1 ml assay buffer (DMEM/F12 no phenol red +0.1% BSA) per paired testes. The cells were counted and diluted to 5 × 10^6^ cells/ml, and 50 ul of this cell suspension was added to each well of a 96-well plate. Testes obtained from five 21 day old rats generally yielded 1 × 10^8^ cells. Cells were incubated with hCG and compounds for 3 h at 37°C in 5% CO2 incubators on a shaker (450 rpm). At the end of incubation, media was collected and testosterone measured by ELISA (EIA‐1559, DRG).

### Human Granulosa Cell Estradiol Production as a Translational Biomarker of Follicular Function

Human granulosa cells were prepared from the spent follicular fluid of women undergoing IVF treatment at Boston IVF Center (Waltham, MA, United States) and at Family Fertility Center (Baylor College of Medicine, Houston, TX, United States) following methods previously established (Breckwoldt et al., 1996; Amsterdam et al., 2003). Institutional review board approval and informed consent from the patients were obtained before collecting the follicular fluid samples. In brief, red blood cells (RBCs) in the follicular aspirate were lyzed using RBC lysis buffer (Quality Biological Inc., Gaithersburg, MD, United States), and washed extensively with PBS and DMEM/F12 containing 5% FBS, fungizone, and the antibiotics penicillin (100 IU/ml) and streptomycin (100 μg/ml). Subsequently, cells from the aspirate were plated in T75 flasks, cultured for 7 days with fresh media being replaced every other day. At the end of the culture period, cells were released from the flask by trypsin and transferred to 96-well plates (5,000 cells/well, Greiner Bio-One). Following 48 h of culture, media in culture wells was replaced with serum free media containing 0.1% BSA and 10^−7^ M androstenedione. Following 24 h of serum starvation, cells were stimulated with fresh media (0.1% BSA, 10^−7^ M androstenedione) with FSH or compound for 48 h. Supernatant was collected and stored at −80C for determination of estradiol by ELISA.

Clinical definitions of patient sub-populations for patients at Texas Fertility followed guidelines outlined by the Practice Committee of the American Society for Reproductive Medicine (Practice Committee of the American Society for Reproductive Medicine, 2015). Normal ovarian reserve (NOR) was defined by day 3 FSH (less than 10–20 IU/L) and estradiol levels within the normal range (less than 60–80 pg/ml), with an anti-mullerian hormone (AMH) value within the normal range for women of comparable age, and a normal antral follicle count (three to eight per ovary). Advanced reproductive age (ARA) was defined as woman of 35 years old or greater in age, based on the definition of advanced maternal age by Society for Maternal-Fetal Medicine. Polycystic Ovarian Syndrome (PCOS) is a patient with two out of the three Rotterdam criteria (Rotterdam ESHRE/ASRM-Sponsored PCOS Consensus Workshop Group, 2004) which includes hyperandrogenism (hirsutism or biochemical), oligomenorrhea or amenorrhea, and/or polycystic ovaries on ultrasound.

### Efficacy and Safety of TOP5300 and TOP5668

#### Phamacokinetics Assays

Pharmacokinetic (PK) studies were performed in female rats by oral (5 mg/kg) or intravenous (1 mg/kg) administration of the compound at ChemPartner (Shanghai, China). Blood samples were collected at serial time points from 5 min to 24 h. The circulating concentrations of the compound in plasma samples were analyzed by HPLC. PK parameters were determined with Phoenix WinNonlin software (Pharsight Corporation, Sunnyvale, CA, United States) using a non-compartmental model.

#### Superovulation Assays

Immature SD rats and B6C3F1 mice (both 18–21 day old females) were purchased from Charles-River laboratories (Wilmington, MA, United States and Shanghai, China). All animals were treated in accordance to Chempartner (Shanghai, China) or Embryotech (Haverhill, MA, United States) IACUC approved animal protocols. The animals were provided water and food ad libitum and housed under 12 h dark and 12 h light schedules.

Immature rats and mice were used to investigate the ability of the small molecules to induce follicular development and support ovulation as described elsewhere (Steelman and Pohley, 1953) with modifications (Sriraman et al., 2014). Briefly, as reference standard, rh-follitropin-α (rec-hFSH [0.29 or 1.16 IU]) was administered as a subcutaneous injection every 12 h for a total of four doses, and r-hCG [1.75 IU] was provided with FSH at first and last injections. At 48 h, an ovulatory dose of hCG [35 IU; intraperitoneal injection] was provided, and 16–18 h later (64–66 h from first injection) animals were euthanized to isolate the ovulated cumulus-oocyte-complex (COC) from the ampulla. The isolated COC’s were treated with hyaluronidase and the total number of ovulated oocytes was determined microscopically.

For oral formulation, TOP5300 and TOP5668 were dissolved in 20% solutol and delivered by oral gavage at 10 ml/kg volume with low dose of FSH (0.29 IU). Rats or mice that received small molecule were also treated with hCG (1.75 IU × 2) and low dose FSH [0.29 IU × 4], following methods we previously established (Sriraman et al., 2014). The low dose of FSH (0.29 IU) provided 20% activity in superovulation assays relative to a concentration of FSH that gave maximum superovulation response (1.16 IU). The ability of TOP5300 to induce ovulation in the absence of low dose FSH was also tested. In some experiments, serum was collected from the animals prior to the ovulatory dose of hCG to determine estradiol levels. Plasma concentrations of FSHR-AA were measured during superovulation assays, except for experiments where plasma was collected for estradiol measurements.

#### IVF and Embryo Development

Developmental competence of oocytes was determined in mice, as described previously (Sriraman et al., 2014). Mice were superovulated with FSH or FSH + TOP5300. Following hCG administration, ovulated COCs were isolated from the oviducts. To measure nuclear status, oocytes were assessed for perivitelline space and first polar body extrusion by phase contrast inverted microscopy. Oocyte maturity was determined by presence of a polar body reflecting oocytes in metaphase-II (MII). Oocytes without a first polar body were assessed as metaphase-I (MI). After the evaluations, oocytes were incubated for 2 h in the media before use in IVF as described (Sriraman et al., 2014). Briefly, oocytes were placed in fertilization media (Research Fertilization Media, Cook Medical, Bloomington, IN, United States) under paraffin oil (Calbiochem, San Diego, CA, United States) and incubated at 37°C in 6% CO_2_, 5% O_2_ and 89% N_2_ for 5–6 h with spermatozoa. Spermatozoa were collected from the cauda epididymis of male C57BL/6J mice and were previously incubated for 1 h in fertilization media prior to use (Research Fertilization Media; Cook Medical, Bloomington, IN) for sperm capacitation. The putative zygotes were then washed and placed in 20 μL drops of culture media (Research Cleave Media; Cook Medical, Bloomington, IN, United States) in Petri dishes, or in 60 μL of culture media in Primo Vision microwell group culture dishes (Vitrolife, Gothenberg, Sweden) and checked for fertilization/cleavage the next morning. Embryos were then cultured in the incubator for an additional 3 days to the blastocyst stage and the blastocyst development rate was recorded.

### Absorption, Distribution Metabolism and Excretion (ADME) and Toxicology Evaluation

As part of the drug development effort, TOP5300 was evaluated in standard ADME, including Cytochrome P450 inhibition, clearance and pharmacokinetic profiles of the compounds. Toxicological evaluations were performed in both rat and dog as the second species according to the guidance from FDA. TOP5300 was suspended in 0.5% CMC-Na and administered for 14 days (Medicilon, Shanghai, China), to male (217.6 to 323.4 g) and female Sprague Dawley rats (170.8 to 211.6 g), housed individually in stainless steel wire-mesh type cages with food and water available ad libitum. The animals were administered vehicle or 30, 100, 300 and 1,000 mg/kg by oral gavage for 14 days. Each treatment group had *n* = 3 animals/sex. Separate groups of animals (n = 6/sex/dose) were used for toxicokinetic (TK) analysis. On day 1 and day 14 of drug administration, serial blood samples were collected from 5 min to 24 h from the TK group animals for analytical measurement of the compound. Blood samples were placed on ice immediately after collection and were centrifuged at approximately 8,000 rpm for 6 min at 2–8°C and the resulting plasma were separated and stored in pre-labeled plastic tubes or vials. The amount of compound in the plasma samples were measure using an HPLC method. All animals were observed for morbidity, mortality, injury, signs of pain or distress, and availability of food and water twice daily (a.m. and p.m.) during the acclimation and observation phase. Evaluated parameters included; mortality, clinical signs, body weight, food consumption, urinalysis, hematology, clinical chemistry, gross post mortem examinations, organ weights and histopathological evaluations of selected tissues. At the end of 14-days of treatment, animals were sacrificed and blood collected for serum chemistry, coagulation and hematological analysis, organ weighed and histological analysis performed in select tissue from control and high dose group.

Male and female beagle dogs were received from Beijing Marshall Biotechnology Co., Ltd (Beijing, China) and group housed at Covance (Shanghai, China), except for 5 h each day in stainless steel cages for study-related procedures. TOP5300 was micronized to improve oral bioavailability and solubilized in the vehicle (0.25% (w/v) carboxymethylcellulose sodium (CMC-Na)) in deionized water and administered by oral gavage once daily for 14 days at a dose volume of 10 ml/kg at 30 mg/kg, 100 mg/kg or 1,000 mg/kg (*n* = 2/sex/dose). On day1 and day 14 of drug administration, serial blood samples were collected from 5 min to 24 h following drug administration, for analytical measurement of the compound for TK analysis. Blood samples (approximately 1.0 ml) were collected for thyroid hormone analysis on Days 0, prior to dosing, 24 h post dose (prior to second dose on Day 2) and once prior to dosing on Day 14. Blood was collected into serum separator tubes (without anticoagulant), allowed to clot at room temperature, and centrifuged for approximately 10 min in a refrigerated centrifuge at approximately at 3,500 g within 2 h of collection. Serum was harvested and placed in a freezer for subsequent thyroid hormone measurement. All animals were observed for morbidity, mortality, injury, signs of pain or distress, and availability of food and water twice daily (a.m. and p.m.) during the acclimation and observation phase. Evaluated parameters included; mortality, clinical signs, body weight, food consumption, urinalysis, hematology, clinical chemistry, gross post mortem examinations, organ weights and histopathological evaluations of selected tissues. At the end of 14-days of treatment, animals were sacrificed and blood collected for serum chemistry, coagulation and hematological analysis, organ weighed and histological analysis performed in select tissue from control and high dose group.

### Data Analyses and Statistics

The results are provided as Mean ± SD from multiple independent experiments (provided in text or figure legends). Data was analyzed using Microsoft Excel and GraphPad Prism (San Diego, CA, United States) for statistical significance by performing ANOVA followed by the Neuman-Keuls test. *p* values less than 0.05 were considered statistically significant.

## Results

### TOP5300 and TOP5668 Were Identified Among 700 Compounds Synthesized in a Hit-To-Lead Campaign for FSH Allosteric Agonists

**FIGURE 1:**
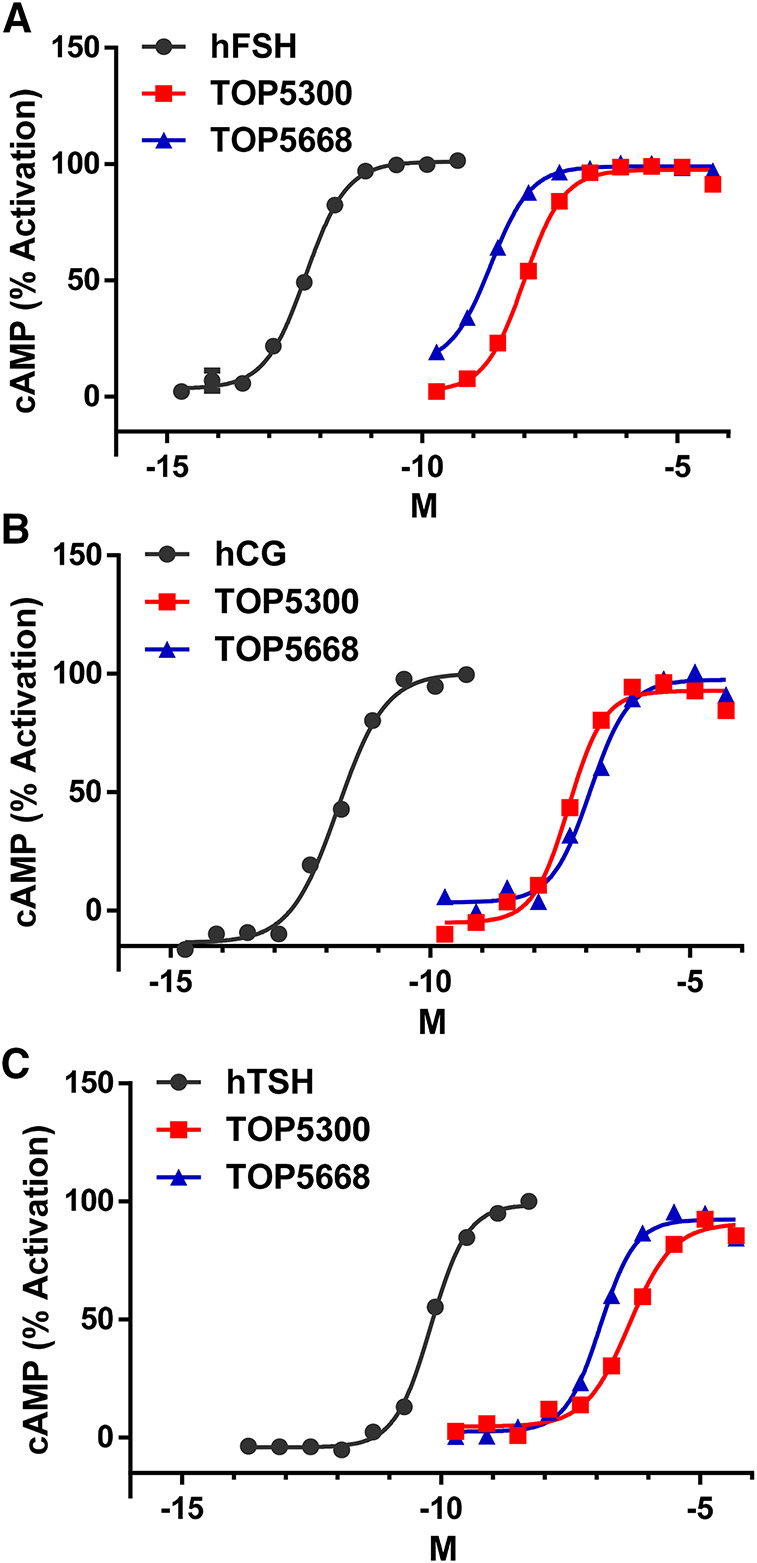
Dose response of TOP5300 and TOP5668 in CHO cells transfected with gonadotropin receptors. Ligand induced cAMP was measured in CHO cells expressing FSH receptor **(A)**, LH/CG receptor **(B)** and TSH receptor **(C)**. The compounds were stimulated in the presence of EC20 ligand in each cell system. Data is representation of one experiment. Each compound was evaluated with different batches of compound synthesized and in multiple experiments as provided in Table 1.

The project to identify small molecule allosteric agonist for FSHR started with high throughput screen (HTS) of about 0.75 million compounds using CHO-K1 cells expressing the hFSHR (CHO-FSHR cells). In the early stages of this program, compounds were evaluated in CHO-FSHR in the presence of a very low concentration of rec-hFSH (0.16 pM) to select compounds that behave as positive allosteric modulators (PAMs). The purpose of the low concentration of rec-hFSH (0.16 pM) was to reduce the threshold for activation of the FSHR (cAMP production) during the early stages of drug discovery in which lower potency compounds were available. From the HTS several hits were identified. Following a chemistry campaign, hits were optimized, and 700 unique compounds were synthesized at TocopheRx. TOP5300 and TOP5668 (dihydropyrazoles; chemical structures, Supplementary Figure S1) emerged as lead molecules; both demonstrated dose dependent increase in cAMP production (Figure 1A). Similar adaptations for CHO-hLHR (Figure 1B) and CHO-hTSHR (Figure 1C) assays incorporated low concentrations of their respective protein ligands. The dose response of the compounds in CHO-hLHR (Figure 1B) and CHO-hTSHR (Figure 1C) show the difference in the potency of the compound to stimulate glycoprotein hormone receptors (Table 1). Twelve different batches of TOP5300 were analyzed in 26 independent assays to generate the hFSHR-EC50 (9.72 ± 0.74 nM; mean ± SEM- Table 1A) demonstrating reproducible batch consistency. TOP5300 represented a mixed hFSHR-AA and luteinizing hormone receptor (hLHR)-AA, with approximately 6-fold less activity in hLHR activity relative to hFSHR activity (Table 1D). Nine different batches of TOP5668 were prepared and tested in 14 independent assays. TOP5668 (EC50 = 2.2 ± 0.19 nM; mean±SEM Table 1A) was 3-4-fold more potent than TOP5300 in hFSHR-AA activity with minimal detectable hLHR activity (∼80 fold less potent- Tables 1B,D) in CHO-LHR cells. Both TOP5300 and TOP5668 showed 80-fold less activity in hTSHR (Tables 1C,D).

**TABLE 1:** Effective Concentration 50 (EC50) of compounds and ligands to stimulate cAMP in the respective CHO assays.

Ligand	EC50 (M)		% Response	*n*
A. CHO-hFSHR				
hFSH	6.42 + 0.81 E-13		100	16
TOP5300	9.72 + 0.74 E-09		98.4 ± 0.31	26
TOP5668	2.20 + 0.19 E-09		100.75 ± 0.51	14
B. CHO-hLH/CGR				
Ligand	EC50 (M)		% Response	*n*
hCG	9.32 + 1.02 E-13		100	13
TOP5300	5.67 + 0.50 E-08		95.68 ± 0.37	18
TOP5668	1.76 + 0.34 E-07		96.64 ± 0.61	8
C. CHO-hTSHR				
Ligand	EC50 (M)		% Response	*n*
TSH	8.27 + 1.18 E-11		100	16
TOP5300	5.05 + 0.05 E-07		84.97 ± 0.85	26
TOP5668	1.70 + 0.20 E-07		92.28 ± 0.88	14
D. Ratio of EC50 from LHR/FSHR and TSHR/FSHR				
Ligand	LHR/FSHR	TSHR/FSHR		
TOP5300	6	52		
TOP5668	80	77		

Percent response is the maximal cAMP induced by the small molecule ligands compared to maximal response of the protein ligands (FSH or hCG or TSH). Data is mean ± SEM for both EC50 and % response. n = independent experiments with duplicate measurements in each experiment to generate EC50 and % response.

### TOP05300 and TOP05668 Stimulate Estradiol Production in Rat Granulosa Cell Cultures, While Only TOP5300 Induces Testosterone Production in Leydig Cells

**FIGURE 2:**
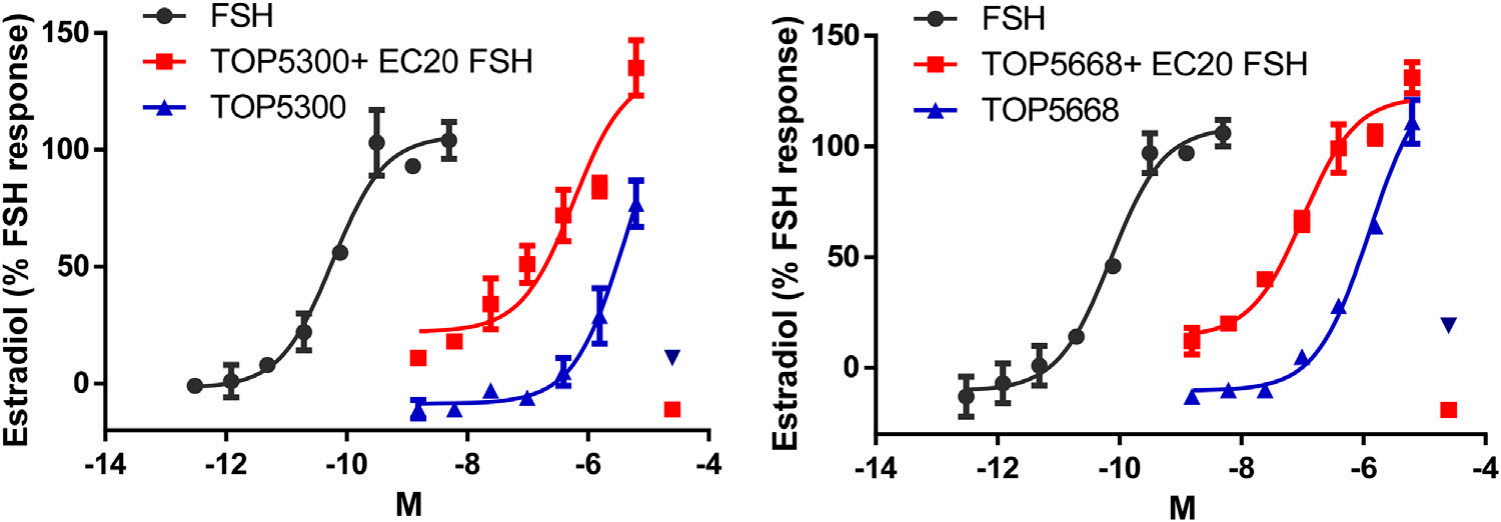
Dose response of TOP5300 and TOP5668 in rat granulosa cell cultures in the absence or presence of a low concentration of FSH (EC20 = 0.20 pM). Rat granulosa cells were stimulated with varying doses of the compounds or FSH for 24 h and estradiol in the media measured by ELISA. Data is Mean ± SD, *n* = two to three expts.

**FIGURE 3:**
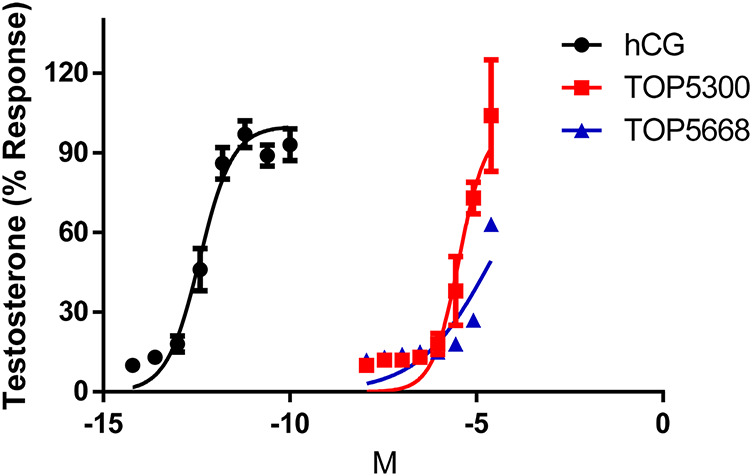
Dose response of hCG, TOP5300 and TOP5668 in rat Leydig cells. Compounds were incubated for 3 h in Leydig cells and testosterone in supernatant measured. Data mean + SD n = 3; (for TOP5668, n = 1), Triplicate determination in each experiment.

In cultured rat granulosa cells obtained from DES-treated rats, TOP5300 (Figure 2A) and TOP5668 (Figure 2B) stimulated concentration-dependent increases in estradiol production in the absence or presence of a low concentration of rec-hFSH (0.2 pM). For both TOP5300 and TOP5668, the EC50 for estradiol production was improved 2-fold by the presence of a low concentration of rec-hFSH. TOP5668 was 5-fold more potent than TOP5300 (Table 2). TOP5300 also stimulated concentration-dependent increases in testosterone production from cultured rat Leydig cells (Figure 3), while TOP5668 showed very little activity in this assay. Allosteric augmentation of the *Lhr* response by TOP5300 in the presence of recombinant LH/hCG was not evaluated in this experiment.

**TABLE 2:** EC50 for TOP5300 and TOP5668 in rat granulosa cell assay measuring secreted estradiol.

	No FSH			+EC20 FSH		
	EC50 (M)	% Response	*n*	EC50 (M)	% Response	*n*
FSH	5.35 ± 1.11 E-11	100	3	5.10 ± 0.47 E-11	100	8
TOP5300	8.68 ± 1.26 E-07	65 ± 6	3	1.75 ± 0.54 E-07	125 ± 12	8
TOP5668	1.82 E-07	93	2	7.33 ± 3.09 E-08	117 ± 8	4

Data is mean ± SEM for both EC50 and % response. n = independent experiments using triplicate determinations at each concentration to generate EC50 and % response.

### TOP05300 and TOP05668 Stimulate Estradiol Production From Human Granulosa Cells, a Disease-Relevant Translational Biomarker of Follicular Maturation

**FIGURE 4:**
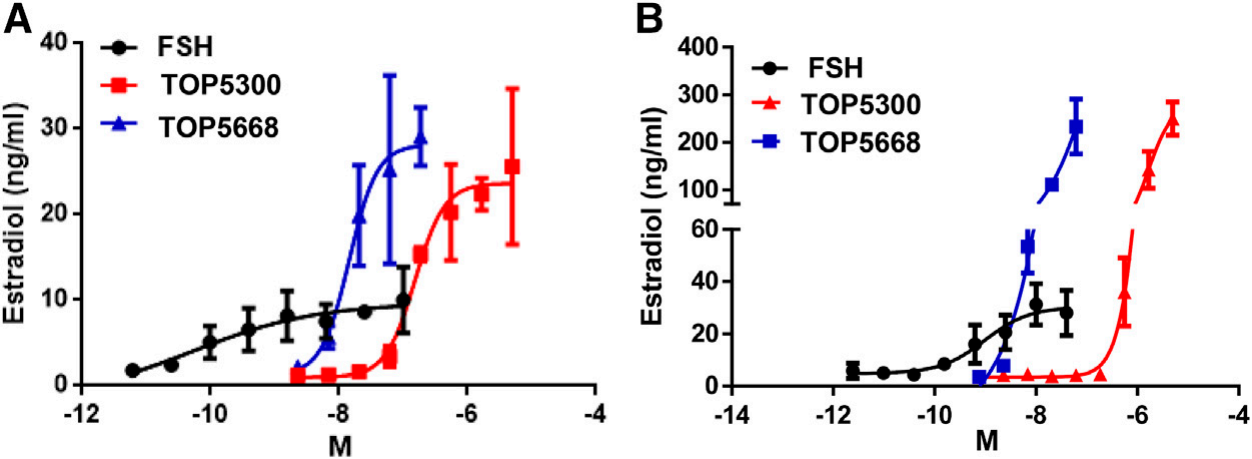
TOP5300 and TOP5668 are full agonists on the FSH receptor in human granulosa cells. Human granulosa cells were obtained from IVF patients immediately following retrieval and processed as described in Methods section for culture. The cells were cultured for 1 week to regain the responsiveness to gonadotropins (van de Lagemaat et al., 2009; Sriraman et al., 2014), and were subsequently challenged with FSH, TOP5300, or TOP5668. Experiment 4A contains pooled granulosa cells from two patients and Experiment 4B contains cells from three patients. Data is Mean ± SD of triplicate determination.

As a first translational assessment, TOP5300, TOP5668 and rec-hFSH were evaluated in human granulosa-lutein cells obtained with consent from patients going through IVF cycle (defined in Materials and Methods), following controlled ovarian stimulation at Boston IVF (Waltham, MA, United States). Follicular aspirates obtained from multiple patients were pooled to insure sufficient cells for testing both TOP5300 and TOP5688 in the same experiments. TOP5668 (exclusive FSHR-AA; EC50 = 15 nM) was 30-fold more potent than TOP5300 (FSHR/LHR AA; EC50 = 474 nM) in stimulating 17β-estradiol production from pooled granulosa-lutein cells obtained from two patients (Figure 4A). Both TOP5300 and TOP5668 achieved substantially greater maximal estradiol production compared to rec-hFSH, at concentrations that were 10-fold to 100-fold higher than that required for rec-hFSH. This potency difference is less than expected from CHO-FSHR results (>1000-fold difference in potency). To confirm these observations, a second culture was performed with pooled granulosa cells obtained from three additional consenting patients (Figure 4B). More dramatic separation of efficacy and potency between hFSHR-AA and recombinant FSH was obtained in the second culture; the potency of TOP5668 was nearly 100-fold shifted to the left of TOP5300, and both compounds achieved a maximal 17β−estradiol response (200 ng/ml) that was approximately 7-fold greater, at a 10-fold higher concentration of TOP agonist than obtained with rec-hFSH. Since the response to FSH varied across different individual patients, and across diagnoses, an EC20 FSH could not be determined, so in these experiments, it was not possible to test for positive allosteric activity.

### TOP5300 Was Distinguished From TOP5668 on ADME/PK (Absorption, Distribution, Metabolism, Excretion/Pharmacokinetic) Properties

Intrinsic metabolic clearance of TOP5300 and TOP5668 was sufficiently stable in rat, human, and dog liver microsomes, and least stable in microsomes obtained from monkeys (Table 3). Neither TOP5300 nor TOP5668 inhibited cytochrome P450 enzymes up to 10 μM (52% of vehicle), nor did either compound contribute to time dependent inhibition of CYP3A4 (Table 3). These results indicate that prolonged exposure to TOP5300 or TOP5668 would not lead to accruing inhibition of the enzyme. In rat, the oral bioavailability of TOP5300 (20%) was higher than TOP5668 (5%) Table 3. On the other hand, in mice it was similar for both compounds (Table 3).

**TABLE 3:** Absorption, Distribution, Metabolism and Excretion (ADME) properties of TOP5300 and TOP5668.

Parameter	TOP5300	TOP5668
Clint (r,h,d,monkey, mice) (µl/min/mg protein)	11,37,37,165,19	7,20,44,117,10
CYP inh @ 10 µM	CYP3A4 (midazolam) = 52%	CYP3A4 (midazolam) = 50.6%
CYP TDI (3A4)	Negative	Negative
*Rat* PK (AUC, T1/2, Cmax, %F)	2,655, 5.1 h, 237, 20% @ 10 mg/kg	1,530, 2.4 h, 132, 5.4% @ 5 mg/kg
Mouse PK (AUC, T1/2, Cmax, F%)	5,533, 2.5 h, 1,133,22% @ 5 mg/kg	2,494, 3.57, 312, 26% @ 5 mg/kg
Dog PK (AUC, T1/2, Cmax, F%)	8,719, 9.4 h, 391,32% @ 10 mg/kg	ND

Clint-Intrinsic Clearance; r-Rat; h-Human; d-Dog; Cyp inh- Cytochrome P450 enzymes inhibition; TDI-Time dependent inhibition; PK- Pharmacokinetics; AUC- Area under the curve (ng*h/ml); T1/2- Time for clearance of half the compound in circulation (h); Cmax Maximal exposure (ng/ml); %F- % oral bioavailability; ND- not determined.

### TOP5300 and TOP5668 Stimulate Follicular Growth *In Vivo* in Immature Rats

**FIGURE 5:**
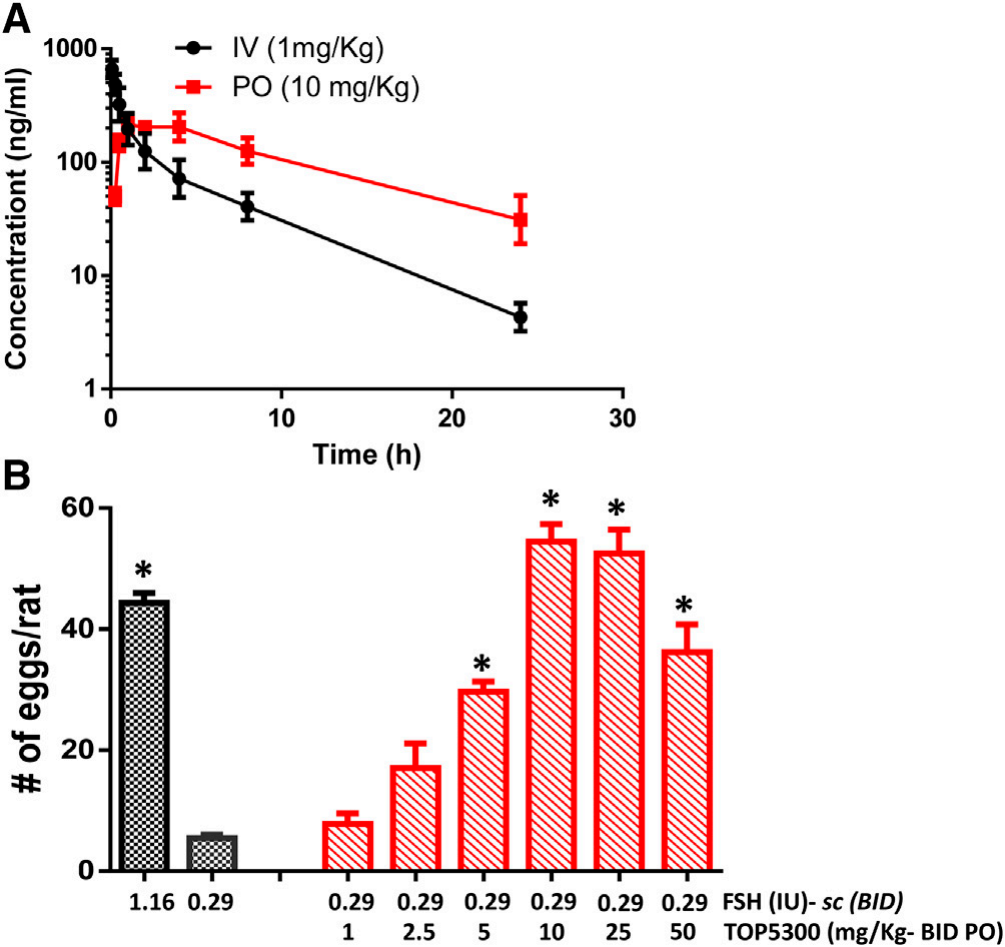
*In vivo* effect of TOP5300 in immature rat. **(A)**. Pharmacokinetics (PK) of TOP5300 in rat. **(B)**. Dose response of TOP5300 in superovulation model. Data is Mean ± SEM, *n* = two to five expts, with five animals/group/expt. *p < 0.05 vs. 0.29 IU FSH, ANOVA.

**FIGURE 6:**
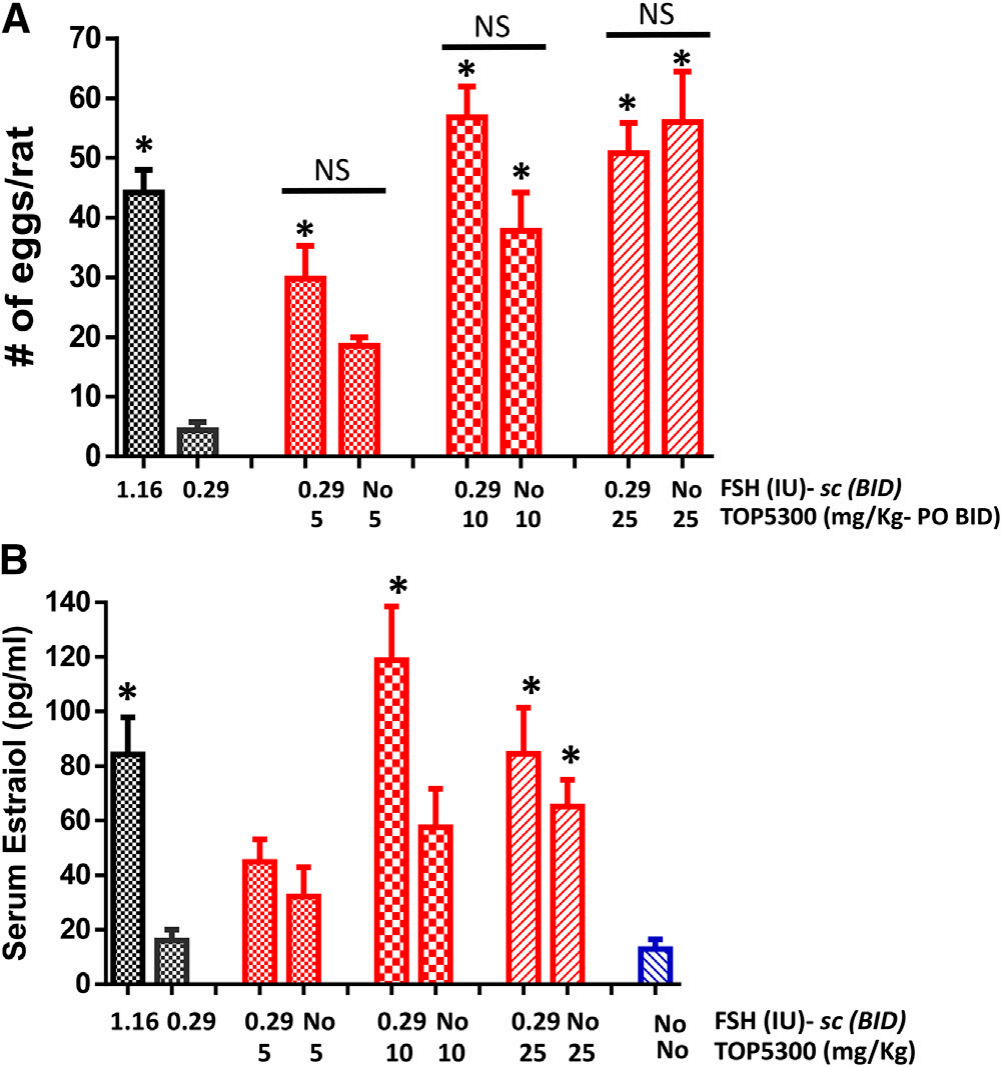
Stimulation of follicular development by TOP5300 ± FSH in immature rat. **(A)**. Oocyte count and **(B)**. Serum estradiol. Data is Mean ± SEM, *n* = 2 experiments with five animals/group. *p < 0.05 vs. 0.29 IU FSH, ANOVA.

The pharmacokinetic profile of TOP5300 demonstrates good exposure by oral administration (Figure 5A). The efficacy of TOP5300 to stimulate follicular development is demonstrated in Figure 5B. TOP5300 in the presence of low dose rec-hFSH was able to stimulate egg production dose dependently (Figure 5B). To provide consistency in the experimental design for TOP5300 and rec-hFSH, both FSHR agonists were administered twice per day; this is the dosing frequency required to observe a response for rec-hFSH in this rat model. The minimal effective dose that generated significantly more cumulus oocyte complex (COC) than saline was 5 mg/kg (Figure 5B). At 10 mg/kg, and 25 mg/kg, the number of COCs obtained was similar to the number of COCs with a maximally-effective dose of rec-hFSH (1.16 IU; total dose over four injections) (Figure 5B). At 50 mg/kg, the number of COCs released by TOP5300 remained significantly higher than 0.29 IU rec-hFSH, and was not significantly different from 1.16 IU rec-hFSHH (Figure 5B). A separate set of experiments was conducted to evaluate the need for low dose rec-hFSH with TOP5300 to stimulate folliculogenesis. Immature rats received TOP05300 with or without supplemental rec-hFSH (0.29 IU) (Figure 6A). TOP5300 is a full agonist that can achieve the same level of follicular maturation on its own or in the presence of a 0.29 IU rec-hFSH (Figure 6A). In these treatment groups, estradiol-17β production followed the follicular stimulation response from 5, 10 and 25 mg/kg doses (Figure 6B). The number of COCs released and serum estradiol-17β levels were not significantly different between vehicle-treated immature animals and the low dose rec-hFSH (0.29 IU). Unlike rec-hFSH, a single daily dose of TOP5300 (morning) was equally effective as twice daily administrations in stimulating follicular maturation sufficient for ovulation with hCG (Supplementary Table S1).

**FIGURE 7:**
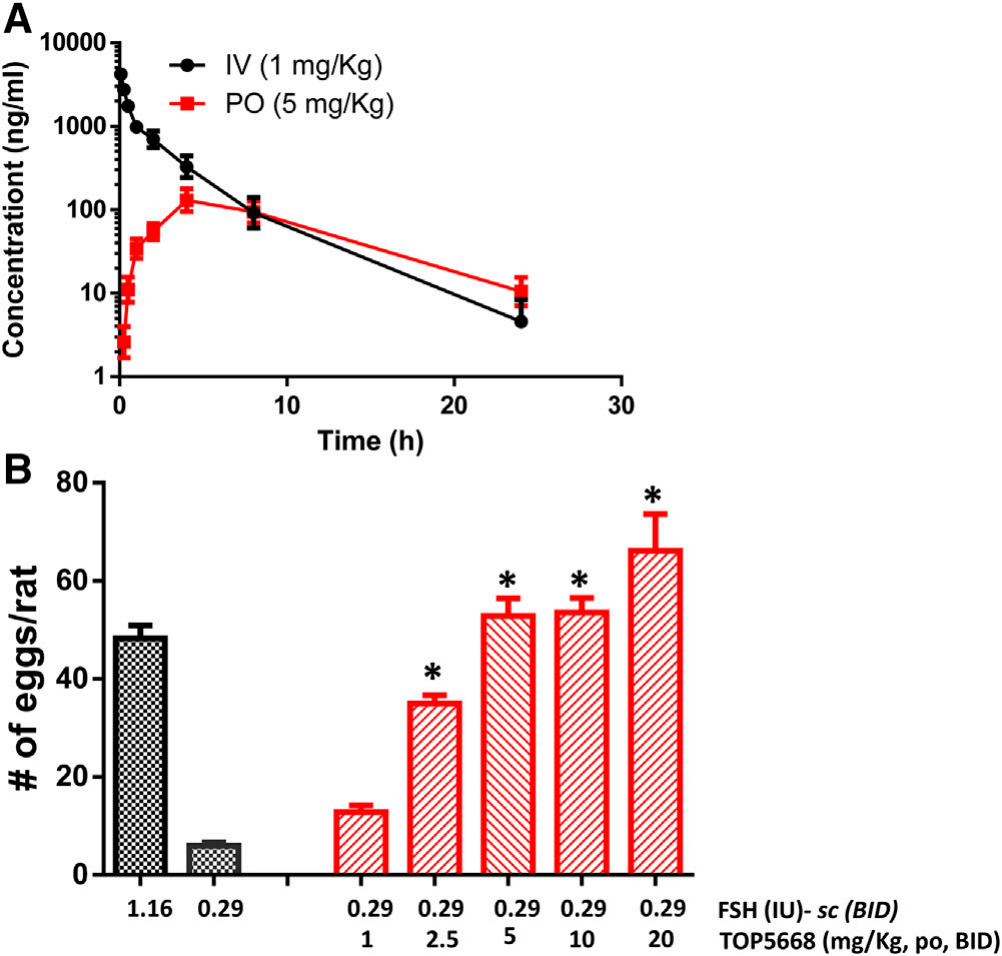
*In vivo* effect of TOP5668 in immature rat. **(A)**. Pharmacokinetics (PK) of TOP5668 in rat. **(B)**. Dose response of TOP5668 in superovulation model. Data is Mean ± SEM, n = one to three expts, with five animals/group/expt. *p < 0.05 vs. 0.29 IU FSH, ANOVA.

TOP5668 (5 mg/kg, oral, Figure 7A) achieved slightly lower exposure in immature rats than was achieved with 10 mg/kg TOP5300 (Figure 6A). The minimum effective dose for TOP5668 in stimulating follicular growth and maturation was 2.5 mg/kg and at higher doses of TOP5668 (5, 10, 20 mg/kg) the number of COCs retrieved increased, reaching plateau at 5 mg/kg with further slight increase at 20 mg/kg (Figure 7B). The dose response curve for TOP5668 indicated that it was approximately 2-fold more potent than TOP5300, despite 3-4-fold lower exposure in mature rat pharmacokinetic models (Table 3). TOP5668 was 14-fold more soluble than TOP5300 in PBS (pH 7.4). Improved solubility in the oral vehicle may have overcome its reduced absorption kinetics and facilitated greater exposure to TOP5668 in the ovarian stimulation assays in immature rats. Due to limitation in resources to pursue both molecules, TOP5668 was designated the backup molecule, so safety pharmacology, toxicology and embryo development studies were done only with TOP5300.

### TOP05300 Exhibits Desirable Safety Profile on Oocytes

**FIGURE 8:**
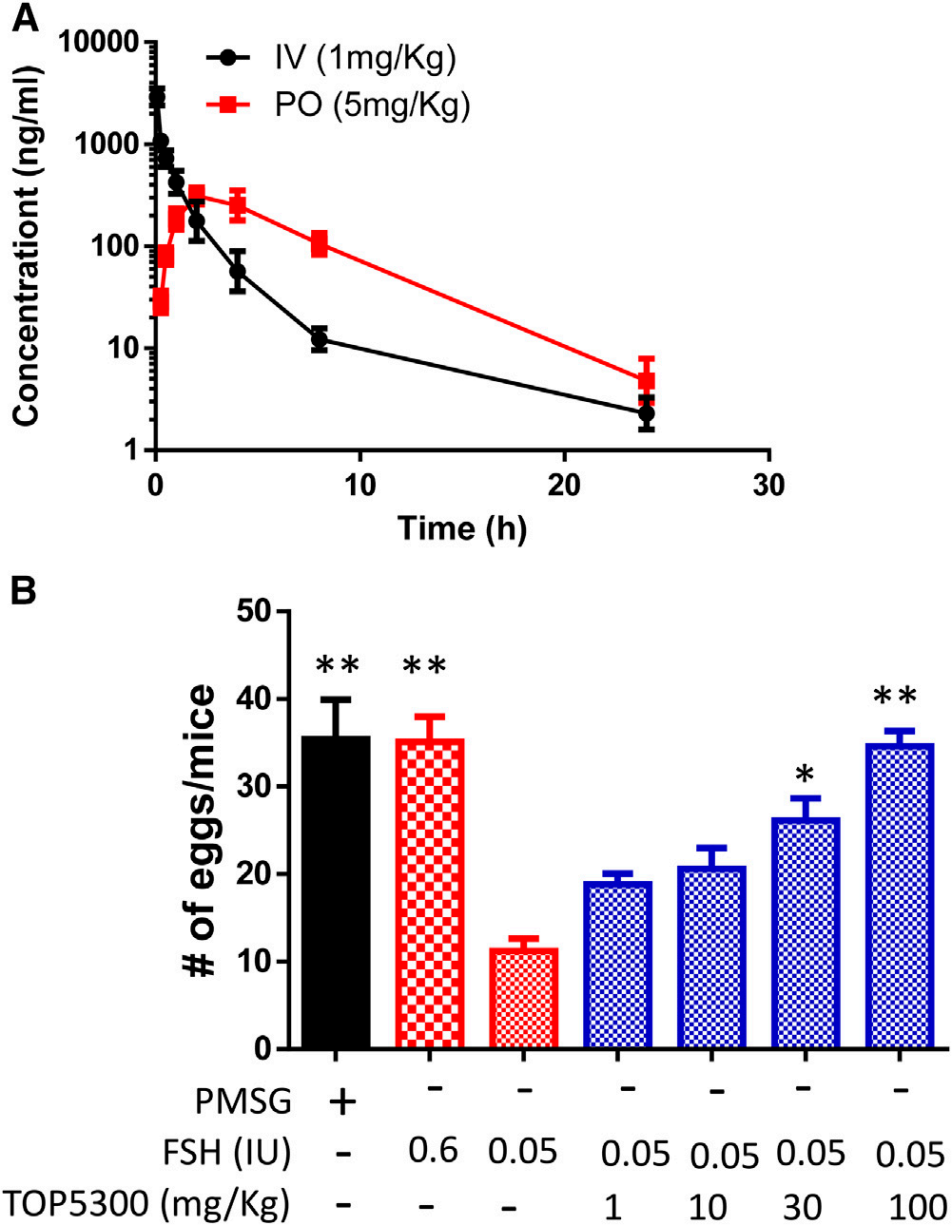
*In vivo* effect of TOP5300 in immature mice. **(A)**. Pharmacokinetics (PK) of TOP5300 in mice. **(B)**. Dose response of TOP5300 in superovulation model in mice. Data is Mean ± SEM, *n* = one to two expt, with five animals/group/expt. *p < 0.05; **p < 0.001 vs. 0.05 IU FSH, ANOVA.

**FIGURE 9:**
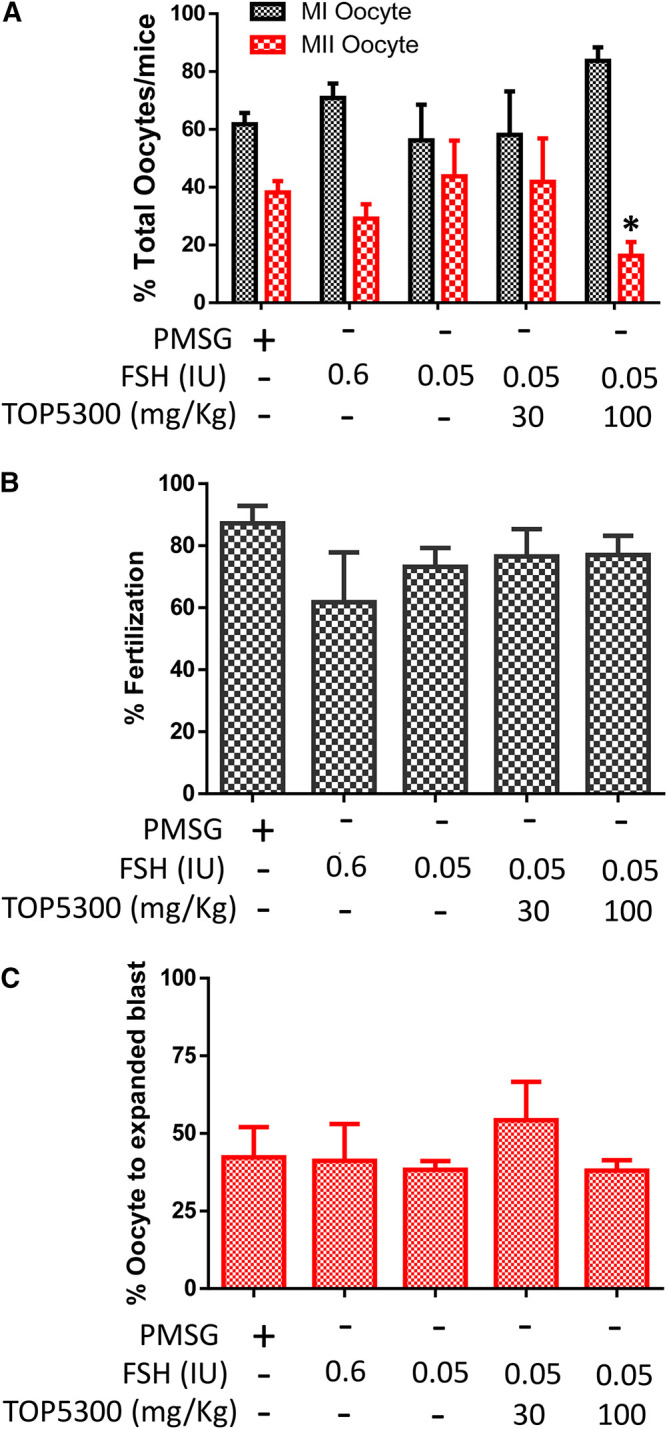
Effect of TOP5300 on oocyte quality, fertilization and embryo development in mice. **(A)**, M1/M2 oocytes; **(B)**. fertilization rate and **(C)**. % expanded blastocysts. Data is Mean ± SD of *n* = 5 animals/group, *p < 0.05, ANOVA, compared to PMSG.

To provide preclinical evidence that TOP5300 was safe for use in ovarian stimulation we evaluated the ability of oocytes exposed to TOP5300 or rec-hFSH to develop into blastocysts. First the exposure of TOP5300 in mice was evaluated in a PK study (Figure 8A). In the mouse model, combined administration of TOP5300 and rec-hFSH was employed to retrieve oocytes from the oviduct to mirror the clinical situation where there is basal levels of FSH in women going through fertility treatment. TOP5300 stimulated follicular maturation at higher doses than used in the rat model, aware that the exposure and half-life of TOP05300 was likely to be lower in mice than in rats, based on experimentally-determined half-life (Table 3). Our adapted dosing achieved slightly greater plasma concentrations of TOP5300 in mice than was anticipated (Figure 8A), and resulted in dose-dependent increases in follicular growth and ovulation at 30 and 100 mg/kg among mice receiving low dose of rec-hFSH (0.15 IU) (Figure 8B). At 100 mg/kg of TOP5300, the ovulatory response was similar to the effect achieved with the high dose of rh-FSH (0.6 IU). Oocytes recovered from the oviduct of treated mice (TOP5300, 30, 100 mg/kg) were recovered, and evaluated for fertilization rates and blastocyst development rates, relative to oocytes obtained from mice treated with rec-hFSH or pregnant mare serum gonadotropin (PMSG; equine chorionic gonadotropin), the traditional positive control for this fertilization experiment (Figure 8B). At 30 mg/kg TOP5300 with low dose FSH, % of M2 oocytes were comparable to FSH and PMSG (Figure 9A). At 100 mg/kg TOP5300, fewer mature oocytes (M2 oocytes) and more M1 oocytes were observed relative to FSH and PMSG group (Figure 9A). Despite the reduction in M2 oocytes at high dose of TOP5300, among all oocytes recovered (M1 + M2) from rec-hFSH-treated or rec-hFSH + TOP5300-treated mice, there were no differences in the fertilization rates (Figure 9B) or hatched blastocyst rates (Figure 9C). This could be due to the fact that the oocytes are incubated for 2 h prior to fertilization and during this period there is maturation of the M1 oocyte. Our results with mice indicate that the oocytes retrieved from TOP5300-stimulated follicles were viable and equally capable of developing into competent blastocysts. These results provide demonstration of acceptable safety profiles for TOP5300, relative to rh-FSH and PMSG, and compatible with development of oocytes and blastocysts for ovarian stimulation regimens in women.

### TOP5300 Meets *In Vitro* and *In Vivo* Safety Profiles to Advance to First-In-Human Studies

The safety and selectivity of TOP5300 were evaluated in an extended panel of assays at Eurofins (Cerep, France, Supplementary Figure S2). In traditional *in vitro* safety assays, TOP5300 was inactive in the potassium (K+) ion channel in cardiac myocytes (hERG assay), in chromosomal genotoxicity assays (Ames assay) and mammalian cell mutagenicity assays (MNT, micronucleus test). TOP5300 was evaluated in 11 phosphodiesterase assays to eliminate the possibility that TOP5300 provided false positives (increase cAMP or cGMP by preventing their hydrolysis) in preclinical experiments (Supplementary Table S1). TOP5300 failed to inhibit activity of a safety panel of 211 kinases, and did not inhibit binding of known ligands in 52 of 55 G-protein coupled receptor assays in this safety panel (Supplementary Table S1). Among the three targets which had >60% activity at 10 uM, dose response analysis was performed. TOP5300 exhibited over 400-fold selectivity against Adenosine A3 receptor, NK2 receptor, and Cl-channel (GABA-gated) (Supplementary Table S1), In a rat toxicology evaluation of TOP5300, all animals survived the duration of the study; there were no effects on mortality. No abnormal clinical signs were observed in this study. At the end of the dosing period, there were no toxicity changes in hematological and coagulation parameters associated with the test substance (Data not shown). Toxicokinetic (TK) observed on day1 and day14 is provided in Supplementary Figure S3. The *T*_max_ of TOP5300–07 was within the range of 2 ∼ 8 h on Day1, and 2 ∼ 8 h on Day14. Increases in AUC_(0–24h)_ and *C*_max_ were proportionally lower than increases in dose on Day 1 and Day 14 in both males and females (Supplementary Figure S3). A gender difference of AUC_(0–24h)_ and *C*_max_ values between the male and female was not observed. Accumulation was not observed in the rats after 14-days repeated dosing of TOP5300–07 once daily for the four dosage groups (Supplementary Figure S3). In females, thymic weight was decreased at 1,000 mg/kg (0.246 ± 0.02 g) compared to vehicle (0.439 ± 0.18 g). Polycystic follicles in the ovary and estrogen-stimulated hyperplasia in the mammary gland, and thymic weight reduction were observed at 1,000 mg/kg. All this is related to the pharmacodynamic effects mediated by FSHR activation. In males, there was no significant findings in any parameters up to 1,000 mg/kg. Based on these findings, the no observed adverse effect limit (NOAEL) is 300 mg/kg in female and 1,000 mg/kg in male.

In the 14-days toxicity assessment of TOP5300 in Beagle dog, no mortality or morbidity occurred. No TOP5300-related effect was noted in clinical observations, body weights, or food consumption. No significant difference was noted in TSH, free T3 or free T4 concentrations over the course of treatment (Supplementary Table S2). Similar effect was also noted in total T3 and total T4 (data not shown). TOP5300 administration had no obvious or adverse effects on hematology, coagulation, and clinical chemistry test results at the terminal sacrifice. The no observed adverse effect level (NOAEL) in dogs in this study was 1,000 mg/kg/day, corresponding to C_max_ values of 2,110 and 1,250 ng/ml, and AUC_0-24_ values of 40,300 and 22,600 h*ng/mL, for male and female dogs, respectively, on Day 14 of the dosing phase.

### Identification of Patient Populations That May Benefit From TOP5300

**FIGURE 10:**
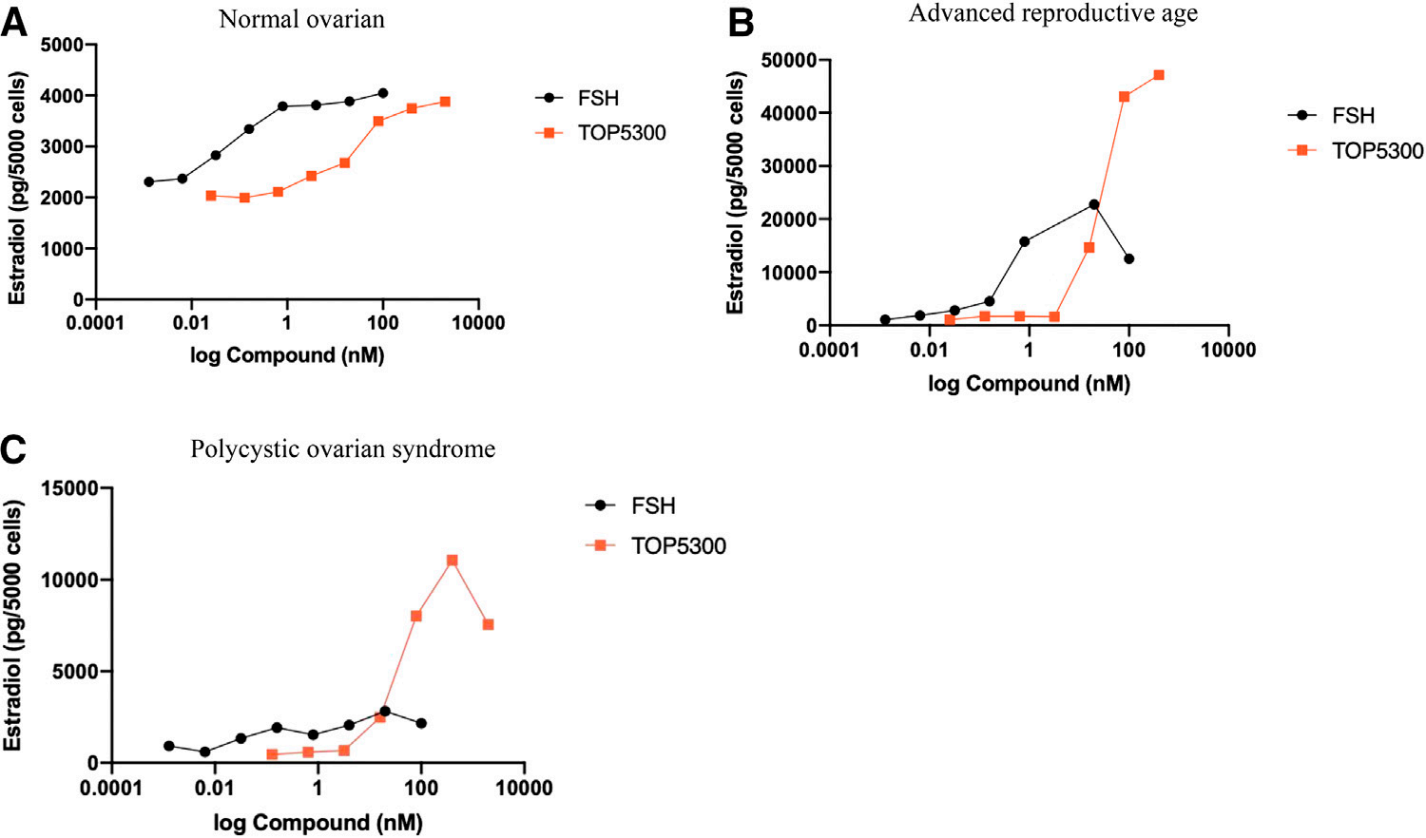
Estradiol production by human granulosa cells from patients with varying clinical manifestation. Granulosa cells obtained from patients with normal ovarian reserve (NOR; 10A), advanced reproductive age (ARA; 10B) and polycystic ovarian syndrome (PCOS; 10C) were stimulated with hFSH (0.00128 to 100 nM) or TOP5300 (0.0256 to 2000 nM). Estradiol in the supernatant was measured by ELISA. Data is representative of one experiment for each diagnostic subset.

The prior translational observations that TOP5300 stimulated estradiol production, similar to rec-hFSH in human granulosa-lutein cells (at Boston IVF; *n* = 2 experiments; pooled granulosa cells) were expanded at the Baylor College of Medicine Center for Drug Discovery, using granulosa-lutein cells obtained by consent from patients at Texas Children’s Hospital Family Fertility Center (Houston, TX; *n* = 12 patients). The diagnosis of infertility was recorded for each patient, and granulosa-lutein cells from individual patients were cultured. Since TOP5300 was identified as the lead candidate for clinical development only this molecule was evaluated in these studies. The results of our pilot study (Figures 10A–C; Table 4) indicated that the concentration of TOP5300 required to stimulate maximum estradiol production was approximately 20-fold higher than rec-hFSH, a difference in potency that was lower than expected based on results with CHO-FSHR or rat granulosa cells (*Fshr*), and consistent with results from Boston IVF. There was large variability in estradiol production across both TOP5300 and rec-hFSH treatment groups and among NOR (*n* = 2), ARA (*n* = 6) and PCOS (*n* = 4) patients. Among cells obtained from women with polycystic ovarian disease (*n* = 4) or from women of advanced reproductive age (*n* = 6), TOP5300 stimulated more estradiol production (Table 4; Figures 10B,C) than cells treated with rec-hFSH. By comparison, estradiol production was similar for both TOP5300 and rec-hFSH in cells from women with normal ovarian reserve (NOR, *n* = 2; Figure 10A). These pilot study results suggest that women with PCOS are the most feasible population to establish a unique effect of TOP5300 relative to rec-hFSH, on granulosa-lutein cells, *in vitro.*

**TABLE 4:** Estradiol production by human granulosa cells in response to FSH (20 nM) or TOP5300 (400 nM).

	Estradiol (pg/ml)		
	NOR (*n* = 2)	ARA (*n* = 6)	PCOS (*n* = 4)
FSH	7,089	4,818 + 8,928	613 ± 1,133
TOP5300	4,829	10,648 ± 7,338	1,276 + 1,563

Granulosa cells were obtained from patients (normal ovarian reserve, NOR; advanced reproductive age, ARA; polycystic ovarian disease, PCOS) seeking IVF treatment. Data are expressed as mean ± SD.

## Discussion

In this report, we have described the preclinical development of two novel FSHR-AA, TOP5300, and TOP5668. TOP5300 incorporates the pharmacologic efficacy of both rec-hFSH and rec-hLH into a single orally active small molecule, while TOP5668 exhibits predominantly rec-hFSH activity. A combination product (rec-hFSH + rec-hLH/rh-CG) has been commonly used by reproductive endocrinologists (Sullivan et al., 1999; Esteves et al., 2009; Thuesen et al., 2012; Deeks, 2018) in controlled ovarian stimulation protocols for over 2 decades. The clinical rationale for combining FSH and LH activity includes the ability of the LH component to moderate stimulation of the single agent rec-hFSH through atresia of small or medium sized follicles, and the potential for LH activity to contribute to high quality oocytes (Macklon and Fauser, 2002; Smitz et al., 2007; Thuesen et al., 2012). On the other hand, many clinicians elect to use injectable products containing predominantly FSH activity. For this reason, two compounds representing either gonadotropin receptor selectivity profile were developed to the point of pre-clinical candidate nomination.

### TOP5300 Is Dramatically Improved Compared to Small Molecules Developed From Our Previous Laboratories (Palmer et al., 2005)

Over the past 23 years since approval of recombinant FSH (1995 in Europe, 1997 in United States), there have been multiple attempts to discover small molecule agonists of both FSH and luteinizing hormone receptors (FSHR, LHR) (Palmer et al., 2005; Yanofsky et al., 2006; Arey et al., 2008; Van Stratten and Timmers, 2009; van Koppen et al., 2013; Gerrits et al., 2016). The key adaptation to our research methods that facilitated the discovery of the dihydropyrazole progenitors of TOP5300 and TOP5668 was the incorporation of a very low dose of rh-FSH (0.16 pM) into our cellular screening assays to reduce the threshold for activation of the FSHR expressed in CHO cells or rat granulosa cells (0.2 pM). This allowed us to identify weak FSHR-PAMs and evolve their activity into allosteric agonists (FSHR-AA) through medicinal chemistry improvements. Among more advanced compounds, this assay format (incorporating low rec-hFSH) in all cellular and *in vivo* formats simulated the low concentration of plasma FSH remaining in plasma of gonadotropin releasing hormone (GnRH)-antagonist downregulated patients. Ultimately, our adapted *in vivo* model measured release of cumulus-enclosed oocytes into the oviduct as a more robust and clinically relevant endpoint of FSHR/LHR-AA activity than changes in ovarian weight (Steelman and Pohley, 1953). Importantly, we demonstrated that addition of rec-hFSH was not required to obtain full agonist activity with TOP5300 in the immature rat ovarian stimulation model. These results indicate that TOP5300 incorporates both FSH/LH agonism and TOP5668 offers FSH agonism without LH activity to suit the various COS protocols used in treatment of female factor infertility.

A second adaptation that facilitated progression of TOP FSHR-AA program was to generate parallel determination of intrinsic metabolic clearance (Clint) for priority compounds (20% of the 700 compounds prepared at TocopheRx) and cytochrome P450 inhibition profiles for high priority (top 1%) compounds selected for further development. None of the compounds described by our group in 2005 had activity in ovarian stimulation models, and measurement of Clint relative to potency was under-utilized at that time (Palmer et al., 2005). Ovarian follicular stimulation for infertility treatments depends on achieving a concentration threshold (Cmax) over the duration of an exposure threshold (AUC) (Macklon and Fauser, 2002).

### TOP5300 Could Be Misinterpreted as Simply a Less Potent Agonist of the FSHR Than Rec-hFSH

The analysis of granulosa cells obtained from individual patients with diagnoses of advanced reproductive age (ARA) or polycystic ovarian syndrome (PCOS: Rotterdam criteria) suggested that TOP5300 elicits enhanced steroidogenic responses in cells from these patients compared with cells from “control” patients with normal ovarian reserve (NOR). TOP5300 and TOP5668 offer an opportunity to explore new mechanisms of FSHR cellular signaling in cell lines and in patient-derived cells. There are receptor internalization and receptor trafficking mechanisms that could be evaluated to further characterize this unique response observed among PCOS patients, and some of these are under investigation with other allosteric agonists. For example, thiazolidinone and benzamide allosteric modulators of the FSHR recruit β-arrestin to FSHR at levels 2-fold higher than rh-FSH (De Pascali et al., 2019; Landomiel et al., 2019). A consequence of greater β-arrestin recruitment by benzamides included an increased level of ERK phosphorylation. Among the same chemical series, a thiazolidinone increased the rate of endosomal recycling by 3-fold, relative to rec-hFSH (Sposini et al., 2020). Recently, a fluorescently-labeled *m*-DHP-Cy5 ligand (Hoogendoorn et al., 2020) was prepared based on the dihydropyridine scaffold (van Koppen et al., 2013) and used to show that FSHR internalization was the same for the DHP chemical series and rec-hFSH. Internalization of either ligand, bound to receptor, was inhibited by a thiazolidinone antagonist (Bonger et al., 2009). An alternative mechanism includes receptor trafficking with insulin-like growth factor receptors (IGF-1R) that have been shown to cooperate in transmitting intracellular signals through rodent and human granulosa cells (Law et al., 2017). According to this mechanism, TOP5300 may induce unique receptor associations among FSHR and IGF-1R intracellular signaling pathways during receptor internalization (Anjali et al., 2015). Similar scaffolding of TSHR and IGF-1R to enhance TSHR activity has been described (Krieger et al., 2019). Components of the IGF-IR pathway, including IRS1 and IRS2 are expressed at higher levels in granulosa cells obtained from PCOS GCs compared with controls (He et al., 2019), providing accessibility to IGF-1R for this cooperative mechanism.

### TOP05300 and TOP05668 Have Similar Efficacy as Rec-hFSH on Ovarian Follicular Growth in Preclinical Models

Our results in preclinical cellular and animal models demonstrate that TOP5300 is an effective stimulator of follicular maturation in two preclinical models (rat, mouse). The primary model used to assess efficacy was the immature rat model that had been used for over 50 years to characterize the potency of purified or recombinant gonadotropins (Steelman and Pohley, 1953). A significant benefit demonstrated in this model was that TOP5300 could be administered at a reduced dosing frequency than rec-hFSH, even though the half-life of TOP5300 and rec-hFSH were not appreciably different in rats. In the context of convenience, these results support superior convenience of TOP5300 relative to injectable rec-hFSH due to the proposed route of administration and frequency of administration. The objective of the mouse model was to establish that oocytes exposed to TOP5300 were capable of developing into competent blastocysts. The results demonstrated that TOP5300 was as safe as rec-hFSH or PMSG for oocyte maturation, fertilization and embryo development.

According to our pharmacokinetics estimation of the half-life of TOP5300 in rats (5.2 h), mice (2.5 h), dogs (9 h), and monkey PK (12.1 h; data not shown), allometric scaling was used to estimate clearance in humans (0.324 L/H/Kg). At least five half-lives of TOP5300 (150 mg QD) would be cleared (from Cmax = 391 ng/ml to 12 ng/ml) before embryos would be transferred back into patients receiving the very earliest transfers of day 2 embryos. At this reduced level of exposure to FSHR-AA, and the selectivity established for TOP5300, an adverse effect of TOP5300 on embryo implantation is not anticipated. Similarly, for controlled ovarian stimulation for intrauterine insemination, it was estimated by allometric scaling that five half-lives of TOP5300 (75 mg QD) would be cleared (from Cmax = 100 ng/ml to 3–6 ng/ml) by the time the embryo entered the uterus for implantation.

The efficacy, selectivity, and safety of TOP5300 in preclinical models suggests that ovarian stimulation with 75–300 mg doses of TOP5300 may deliver comparable responses as 37.5 to 450 IU recombinant FSH (6-fold increase in dose from COS-IUI to COS-IVF). We demonstrated in rats that there is 60-fold margin of safety between first significant measure of efficacy (5 mg/kg) and NOAEL (300 mg/kg) defined by the on-target evidence of ovarian hyperstimulation (1,000 mg/kg). In the rat *in vivo* model, 0.29 IU rh-FSH was without significant effect on follicular maturation and oocyte release, and a 4-fold higher dose, 1.16 IU, represented a maximum follicular response. The separation between incremental doses of TOP5300 and ovulation of incremental numbers of oocytes suggests that greater precision in desired follicular stimulation may be possible in future use of TOP5300 in controlled ovarian stimulation.

### TOP5300 Safety Profile Anticipates Lower Risk of Failure in Clinical Trials Than Previous FSHR-AA Programs

In previous evaluations, we had demonstrated that a thiazolidinone agonist possessed genotoxic effects (Sriraman et al., 2014) emphasizing the need to insure that TOP5300 was free of genotoxic and mutagenic properties. A first level of safety was established for TOP5300 by results that confirmed the absence of *in vitro* safety liabilities (Committee for Human Medicinal Products, 2017), including hERG evaluations, and exceptional selectivity against a safety kinase panel, and against membrane receptors and ion channels. A second level of safety was established by demonstrating that TOP5300 exhibits good separation between FSHR and TSHR efficacy *in vitro* and *in vivo*. A third level of safety was demonstrated by the separation of efficacy (5 mg/kg) and first suggestion of adverse effect (1,000 mg/kg), that was an on-target response in rat toxicological study.

A fourth level of safety was established by demonstrating good separation between FSHR- and TSHR-dependent responses. The FSHR EC50 for TOP5300 and TOP5668 are comparable with a previously reported FSHR-AA, MK-8389 (human FSHR-AA, EC50 = 4.2 nM, TSH receptor EC50 = 340 nM, TSHR/FSHR = 81) (Gerrits et al., 2016). MK-8389 represented an optimized clinical candidate for ovarian stimulation, however, in human phase one study, MK-8389 failed to induce follicular growth, but rather induced TSHR activity. In preclinical pharmacology experiments, these authors reported MK-8389 had a 10-fold separation between ovarian and thyroid stimulation in monkeys (10-fold separation between the dose of MK-8389 required for folliculogenesis (2.25 mg/kg twice daily) and thyroid follicular cell hypertrophy (23 mg/kg) in monkeys). Unfortunately, this 10-fold level of selectivity did not translate in the human clinical trial. While folliculogenesis was expected at 10–20 mg/day, the biomarkers of follicular development, inhibin B and estradiol were not elevated up to the top dose of 40 mg/day tested in the study. On the other hand, thyroid activity was evident at 20–30 mg/day in women. Although thyroid function parameters returned to baseline within 7 days of discontinuing MK-8389 treatment, the absence of separation between FSHR and TSHR pharmacology led to termination of this development program. Among the explanations considered by the authors were the peak-to-trough variation in MK-8389 levels during a 24-h dosing interval and differences in bioavailability of MK-8389 in ovary compared to thyroid. This hypothesis suggests that metabolism and clearance of MK-8389 favored exposure suited for TSHR activity (Cmax-dependent response) (Neumann et al., 2020) rather than combined concentration and duration of exposure (AUC) thresholds for ovarian stimulation. Another explanation offered by the authors is that monkeys did not provide a reasonable prediction of the FSHR potency relative to TSHR risk in women.

Our alternative hypotheses for the observed thyroid stimulation by MK-8389 and an absence of ovarian response is largely based on the use of contraceptive progestins that altered both pharmacokinetic and pharmacologic mechanisms of MK-8389, and impaired ovarian stimulation without compromising TSHR responses. The clinical trial design for MK-8389 adapted a previous clinical trial design used for corifollitropin (Duijkers et al., 2002; Balen et al., 2004), including use of an oral contraceptive (Marvelon, containing desogestrel and ethinyl estradiol) to achieve endogenous gonadotropin down-regulation in women, and to provide contraception during the investigational drug evaluation in Phase one. A comparison of two prior clinical trials performed with corifollitropin reveals that inclusion of contraceptives (Duijkers et al., 2002) significantly inhibited the ovarian response to corifollitropin compared to the absence of contraceptive (Balen et al., 2004). This information provides strong support for an alternative hypothesis that the ovarian response to MK-8389 was impacted by the inclusion of progestin-containing oral contraceptive in the phase one clinical trial. Further, time-dependent inhibition of CYP3A4 (Lin et al., 2011) associated with thiophene groups integrated into MK-8389, may be responsible for the multiple phase elimination reported for MK-8389(Gerrits et al., 2016). Metabolic clearance of desogestrel by CYP3A4 is known (Korhonen et al., 2004) and disclosed in the Marvelon package insert. Inhibition of CYP3A4 by MK-8389 would be expected to reduce rates of clearance of desogestrel, and cause elevation of blood concentrations of desogestrel (plasma desogestrel levels not reported by Gerritts, *et al.*) (Gerrits et al., 2016) that suppress ovulation (Heikinheimo et al., 1996).

An awareness of the clinical impact of metabolic clearance of allosteric agonists relative to the protein ligand and the impact they will have on ovarian vs. thyroid responses provides a guide for future clinical trials with FSHR-AA. Prepared by the results from the MK-8389 clinical study, the preclinical discovery of FSHR-AAs at TocopheRx minimized the early risk of failure due to poor selectivity against TSHR. Estimation of TSHR risk in dogs enabled the highest exposure possible for TOP5300 in a preclinical model, and the best model to determine risk of thyroid stimulation for TOP5300. Our results with TOP5300 found no evidence to support an effect on the thyrotrope-thyroid axis. In all safety evaluations conducted with TOP5300, it was determined to be sufficiently safe for first-in-human evaluation.

### TOP5300 and TOP5668 Are Expected to Bind to hFSHR Within the Allosteric Binding Pocket

Prior to definition of the allosteric pocket in the FSHR crystal structure, allosterism of ORG214444 was illustrated by failure to activate cAMP production by cells expressing a mutant FSHR (T449 V (van Koppen et al., 2013)). Previous thiazolidinone agonists were inactive in FSHR mutants with transposed TSHR transmembrane domains (Arey et al., 2008), or their activity could be inhibited by thiazolidinone antagonists (Sriraman et al., 2014). Following analysis of ectodomain crystal structure, it appears that allosteric agonists of FSHR are interacting within the general domain formed at the top by extracellular loops and hinge domain, and along the sides by the transmembrane helices. The proposed allosteric binding pocket predicted for the FSH receptor (Jiang et al., 2014a; Jiang et al., 2014b; Ulloa-Aguirre and Zariñán, 2015), shares general features common among the proposed allosteric sites described for the A2a-AR extracellular cleft domain (Caliman et al., 2018) and for β2-adrenergic receptor agonists (Manglik et al., 2015; Liu et al., 2019). Following binding of the allosteric agonist, the release of 1, two or all three monomers (Jiang et al., 2014b) best explains 3-fold increase in binding of the protein ligand, FSH, because small molecules do not face the steric constraints of fully glycosylated FSH for binding to trimers (Duijkers et al., 2002; Balen et al., 2004; Lin et al., 2011). Cellular responses have been anticipated to differ between FSHR-AA and rec-hFSH, (biased signaling) and may affect: i) FSHR residence time on plasma membrane and influence *G*_*s*_ relative to G_i_ signaling, ii) intracellular trafficking of FSHR may influence intracellular signaling through MAPK, and iii) cross-family activation of LH/CGR or TSHR (Ji et al., 2004). The scope of the present work was to identify allosteric agonists that resembled FSH and achieved similar preclinical pharmacology. Neither the precise site of interaction with FSHR, nor the extent of biased agonism have yet been explored for TOP5300 or TOP5668.

### Translational Evaluation of TOP5300 in Patient Subsets

To recommend TOP5300 to advance into clinical trials, it was considered important to identify a patient population that could expect to receive benefit with this new treatment. Prior to this effort, no previous FSHR allosteric agonist program has attempted to identify distinct patient populations that could benefit from this allosteric mechanism of action. Our pilot study results, although not significantly different, suggest that patients exhibiting criteria of PCOS may obtain benefit from treatment with TOP5300. Patient-to patient variability in estradiol production has shifted our focus from media concentration of steroid to evaluate steroidogenic gene expression patterns in cells from patients with PCOS. We acknowledge that a limitation of this *in vitro* culture model, and its ability to predict benefit from ovarian stimulation outcomes, is that cells obtained from patients have already been exposed to ovulatory doses of hCG, and require a period of resensitization to FSH for this cell culture experiment.

In summary, our research identified novel, oral FSHR-AAs that effectively mimic the biological activity of rh-FSH in each cellular or physiologic system we evaluated. Considerable focus was directed toward development of orally-active FSHR-AAs that were highly selective for FSHR, and provided over 50-fold separation between FSHR and TSHR activity. TOP5300 was shown to be safe for oocytes, and free from off-target and potentially adverse effects through the blastocyst stage, demonstrating acceptable freedom from effects through the first segment of reproductive toxicology evaluation, similar to the toxicology effects included in the FDA NDA package for follitropin-α (Serono Laboratories, 2000). TOP5300 (and TOP5668) represent new allosteric agonists with potential for ovarian stimulation in women.

## Data Availability

The original contributions presented in the study are included in the article/Supplementary Material, further inquiries can be directed to the corresponding authors.
