# Erratum: Discovery and Preclinical Development of Orally Active Small Molecules That Exhibit Highly Selective Follicle Stimulating Hormone Receptor Agonism

**DOI:** 10.3389/fphar.2021.672778

**Published:** 2021-03-31

**Authors:** Frontiers Production Office

**Affiliations:** Frontiers Media SA, Lausanne, Switzerland

**Keywords:** follicle stimulating hormone, oral FSHR allosteric agonist, g-protein coupled receptor, infertility treatment, follicular maturation, oocyte viability

An erratum on Discovery and Preclinical Development of Orally Active Small Molecules That Exhibit Highly Selective Follicle Stimulating Hormone Receptor Agonism by Nataraja S., Yu H., Guner J. and Palmer S. (2021). Front. Pharmacol. 11:602593. doi: 10.3389/fphar.2020.602593


Due to a production error, Supplementary Figures S1, S2 were missing, and Supplementary Figure S3 was used in the article instead of Figure 3. Furthermore, the in-text citation for the tables were incorrect. The in-text citations were updated, the corrected Figure 3 appears below, and the supplementary file has been updated.

**FIGURE 3:**
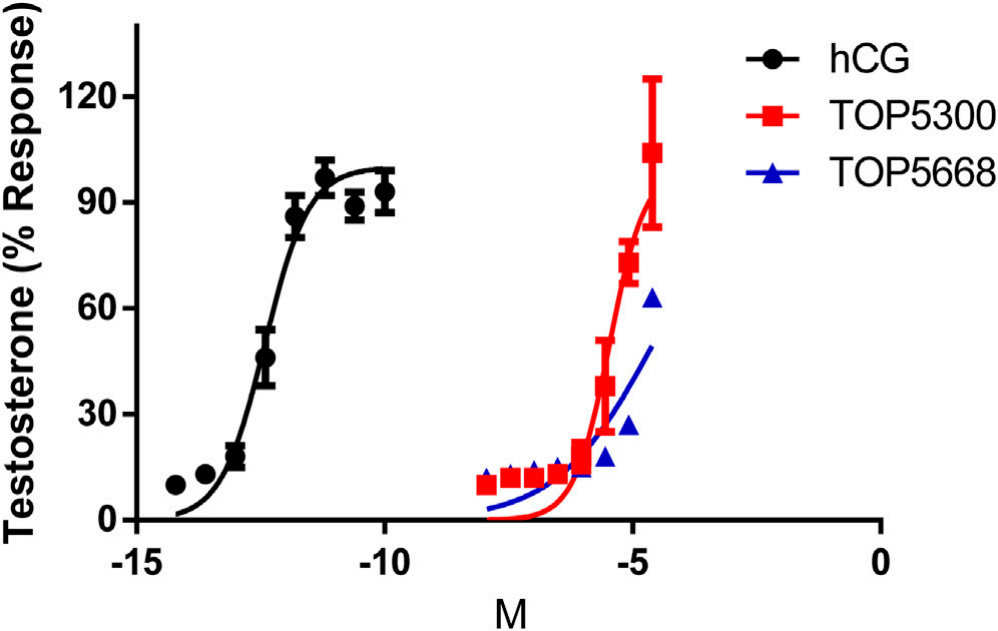
Dose response of hCG, TOP5300 and TOP5668 in rat Leydig cells. Compounds were incubated for 3 h in Leydig cells and testosterone in supernatant measured. Data mean + SD *n* = 3 (for TOP5668, *n* = 1), Triplicate determination in each experiment.

The publisher apologizes for this mistake. The original version of this article has been updated.

