# Supplementary material for: Discovery and Preclinical Development of Orally Active Small Molecules that Exhibit Highly Selective Follicle Stimulating Hormone Receptor Agonism
Source: Front Pharmacol. 2021 Jan 14;11:602593. doi: 10.3389/fphar.2020.602593 (PMC7845544; doi:10.3389/fphar.2020.602593)
Supplement: Supplementary file 1 [file DataSheet1.pdf]

Supplementary Results

Supplementary Figure1 Structure of Compounds

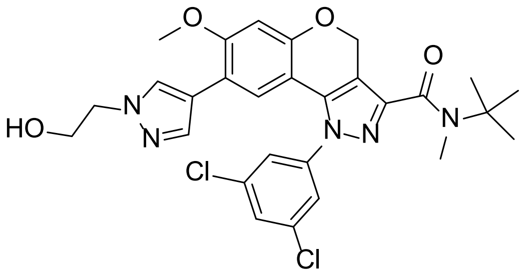

TOP05300

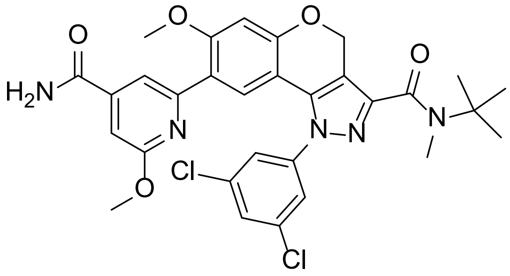

TOP5668

Supplementary Figure 2. TOP5300 stimulates similar follicular growth whether administered twice per day (BID) or once per day (QD) by oral gavage. rh-FSH was administered by subcutaneous injection twice daily. Stimulation of follicular growth was measured by the ability of hCG to induce ovulation of mature follicles and the recovery of cumulus-enclosed oocytes from the oviduct 16-18 hours after injection of hCG. Data is mean±SEM, n=2-3 expts. with 5 animals/group/expt. *p<0.05 vs 0.29IU FSH, ANOVA

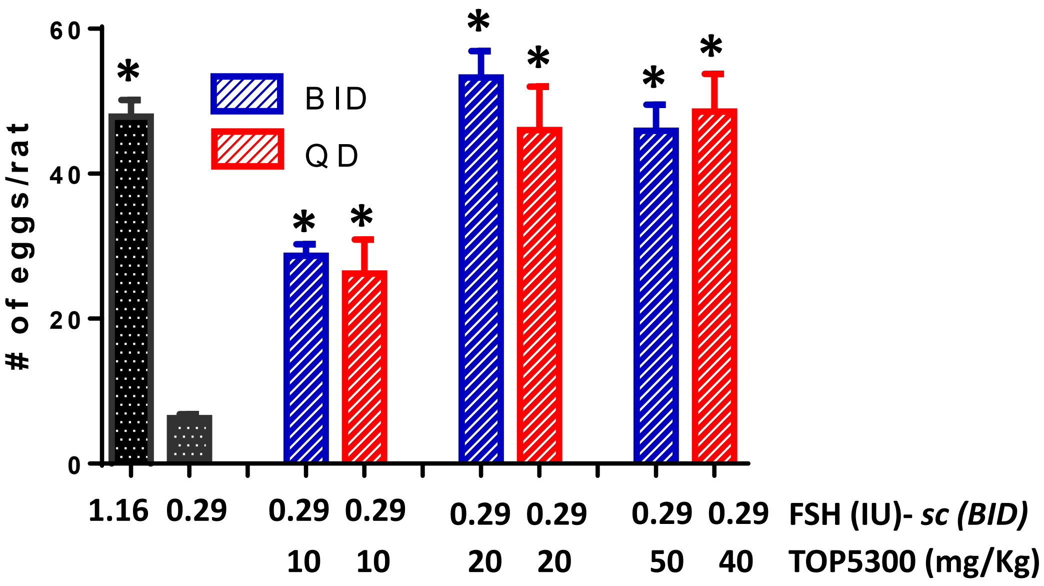

Supplementary Figure 3: Toxicokinetic (TK) profile of TOP5300 in rat treated for 14-days.
Data is mean+SD, n=3 animals/timepoint

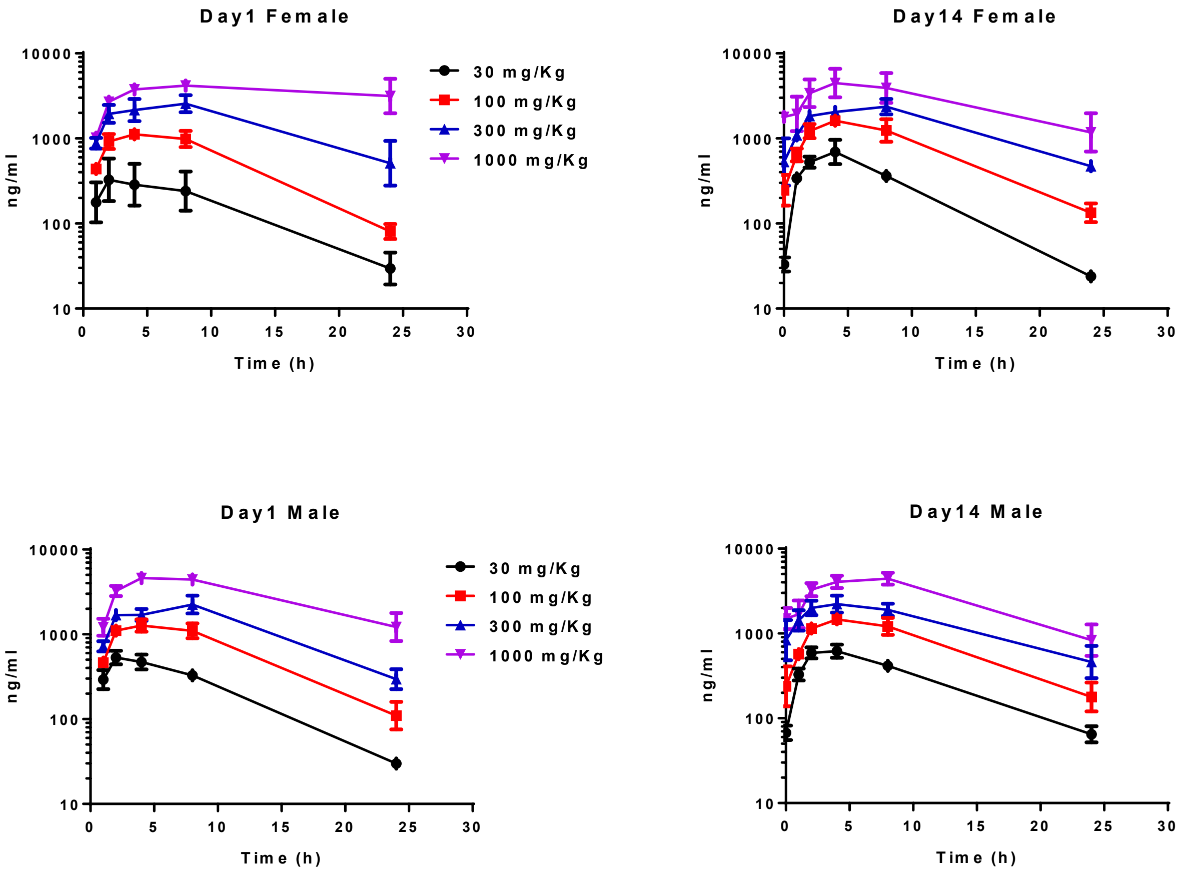

Supplementary Table 1: **Broad safety and selectivity profile of TOP5300.**

Assay name	Assay content	Result
hERG channel inhibition @ 10 uM [Cyprotex]	hERG	16 % inhibition – No significant effect
Gentotoxcity test	Ames test	Negative
Chromosomal Aberrations [Cyprotex]	Micronucleus Test (MNT)	Negative
Phosphodiesterase (PDE) profile @ 10 uM [Eurofins]	Tested against 9 isoforms PDE	No significant inhibition on any isoform
Kinase profile@ 10 uM [Eurofins]	Tested against 211 kinases	No significant inhibition on any target
Single concentration<br>Cerep profile @ 10 uM [Eurofins]	Tested against 55 targets,	52 targets < 60%<br>3 targets > 60% inhibition
Concentration response curve [Eurofins]	Dose response tests against 3 targets (A3(h), NK2(h), Cl- channel (GABA-gated))	Good safety margin compared to FSHR; safety margin > 400 X for all three targets compared to FSHR)

Supplementary Table 2. Serum level of thyroid hormone (Free T3, Free T4 & TSH) in beagle dogs treated with TOP5300 for 14 days, A-female dog and B-male dog. Hormone levels were measured predosing (0hr), 24h after 1st dosing (24h) and 24hr following the 13th dose (Day14). The values are normalized to vehicle treated animals (0 mg/Kg). Data is mean of n=2/sex/dose group.

A

Dose mg/Kg	Free T3				Free T4				TSH		
	Fold change over vehicle group				Fold change over vehicle group				Fold change over vehicle group		
	0h	24h	Day 14		0h	24h	Day 14		0h	24h	Day 14
0	1	1	1		1	1	1		1	1	1
30	0.9	0.8	1.0		1.1	1.1	0.8		1.2	0.9	1.1
100	1.0	0.9	1.1		1.2	1.1	1.1		1.3	1.0	1.1
1000	1.1	0.9	1.2		1.0	0.9	0.9		1.2	0.8	0.8

B

Dose mg/Kg	Free T3				Free T4				TSH		
	Fold change over vehicle group				Fold change over vehicle group				Fold change over vehicle group		
	0h	24h	Day 14		0h	24h	Day 14		0h	24h	Day 14
0	1	1	1		1	1	1		1	1	1
30	1.3	1.3	1.3		1.0	1.0	1.0		0.8	0.9	1.0
100	1.1	1.1	1.1		0.9	0.8	0.9		0.8	0.9	1.0
1000	1.0	0.9	1.0		0.9	0.9	1.0		0.7	0.9	0.8
